# Empowering Recovery: A Co‐Designed Intervention to Transform Care for Operable Lung Cancer

**DOI:** 10.1111/hex.70196

**Published:** 2025-04-05

**Authors:** Georgina A. Whish‐Wilson, Lara Edbrooke, Vinicius Cavalheri, Zoe T. Calulo Rivera, Madeline Cavallaro, Daniel R. Seller, Catherine L. Granger, Selina M. Parry

**Affiliations:** ^1^ Department of Physiotherapy School of Health Sciences The University of Melbourne Melbourne Victoria Australia; ^2^ Department of Health Services Research Peter MacCallum Cancer Centre Melbourne Victoria Australia; ^3^ Curtin School of Allied Health Curtin University Perth Western Australia Australia; ^4^ Curtin enAble Institute, Faculty of Health Sciences Curtin University Perth Western Australia Australia; ^5^ Allied Health, South Metropolitan Health Service Perth Western Australia Australia; ^6^ Department of Physiotherapy The Royal Melbourne Hospital Melbourne Victoria Australia; ^7^ Physiotherapy Team, ORA Therapies, Health New Zealand – Te Whatu Ora, Capital, Coast & Hutt Valley Wellington New Zealand; ^8^ School of Physiotherapy, Northern Centre (Wellington) University of Otago Wellington New Zealand

**Keywords:** co‐design, exercise, lung cancer, prehabilitation, rehabilitation, thoracic surgery

## Abstract

**Background:**

Patients undergoing surgery for lung cancer experience significant symptom burden and physical impairments. Exercise rehabilitation programmes have been shown to improve symptoms and aid recovery, however, implementation into routine practice has proven challenging.

**Objective:**

To develop a robust understanding of the key design requirements of an exercise‐based pre‐ and post‐operative rehabilitation prototype intervention designed to support patients with operable lung cancer prepare for and recover from thoracic surgery, and co‐design an acceptable intervention prototype with key stakeholders.

**Design, Setting and Participants:**

An experience‐based co‐design (EBCD) study involving patients, caregivers, clinicians, consumer advocates and researchers from across Australia. Two rounds of EBCD workshops were held between November 2023 and May 2024. Workshops were underpinned by the COM‐B Model and Theoretical Domains Framework. Qualitative data were thematically analysed by two independent researchers. Identified barriers and facilitators were mapped to the Behaviour Change Wheel, and used to develop the final intervention prototype, which was presented using the Template for Intervention Description and Replication (TIDieR) guide.

**Results:**

Eleven patients (55% female, mean age 66.4 (±9.3) years), one caregiver, and 16 professionals (physiotherapists, nurses, respiratory physicians, a thoracic surgeon, consumer advocates and researchers) participated. Retention between workshop rounds was high (86%). Nineteen major themes were developed, including unmet education needs; the link between mental health and recovery; and the influence of unexpected, persistent symptoms and functional decline. Core intervention principles included flexibility, individualisation and continuity. Essential components included screening/assessment, education, exercise, behaviour change, and mental health support. The intervention prototype was refined in the second workshop round.

**Conclusions:**

This EBCD study successfully identified key experiences and barriers in preparing for and recovering from lung cancer surgery and engaged stakeholders in complex intervention design, culminating in the development of a flexible, multi‐modal pre‐ and post‐operative rehabilitation programme prototype. Future projects will evaluate the prototype acceptability and feasibility.

**Patient or Public Contribution:**

Past patients and their caregivers with lived experience of undergoing/caring for someone undergoing lung cancer surgery, and multidisciplinary professionals, participated in co‐design workshops to develop and refine the exercise‐based rehabilitation intervention goals, priorities, and prototype.

## 1 Background

Patients undergoing lung resection surgery for lung cancer experience a high symptom burden [1]. Common symptoms include pain, dyspnoea, cough, fatigue, sleep and mood disturbances and functional impairments (e.g., reduced mobility, muscle strength and exercise capacity) [1]. Despite the typically curative intent of surgery, symptoms and impairments can persist [1]. Patients may also undergo neoadjuvant and/or adjuvant anti‐cancer treatments such as radiotherapy, chemotherapy, targeted therapy and/or immunotherapy [2]. These treatments can exacerbate post‐operative symptoms and cause additional side‐effects such as gastrointestinal symptoms, anaemia and anorexia [2].

Pre‐ and post‐operative exercise programmes have been shown to improve symptom burden and physical function [3, 4, 5]. Pre‐operative exercise programmes can also reduce the risk of developing post‐operative pulmonary complications and may reduce post‐operative hospital length of stay [3]. Despite these known benefits, the implementation of this evidence into standard lung cancer care has proven challenging worldwide [6, 7, 8]. Internationally, both pre‐operative and post‐operative outpatient exercise programmes are seldom offered as part of routine operable lung cancer care, with the exercise‐based management of these patients typically limited to inpatient post‐operative interventions (e.g., early mobilisation) [6, 7, 8]. In 2023, our research team published a survey of Australian and New Zealand health services and identified only eight pre‐operative and 39 post‐operative exercise programmes available for patients with operable lung cancer [8]. Most of these services were pulmonary rehabilitation programmes, offering in‐person, centre‐based group exercise training [8]. Barriers to implementation include existing workplace culture/practice, limited resources, and the lack of consensus regarding key programme design requirements [8, 9, 10, 11].

Patient and public involvement (PPI) has been identified as pivotal across all aspects of cancer control [12]. Co‐design, a participatory action research approach, is being increasingly utilised to target implementation barriers [13]. Co‐design iteratively considers and incorporates participant perspectives; barriers and facilitators to implementation; and scalability throughout all design stages, resulting in interventions that are likely to be more acceptable and feasible [13, 14]. The terms ‘co‐design’ and ‘co‐production’ are often used interchangeably, with different models existing to distinguish between them [15]. Several of these models have proposed that co‐production uses PPI in the ‘co‐implementation’ of a pre‐determined service/solution to a problem, whereas co‐design involves the collaborative development of a service/solution to a problem through PPI [16, 17, 18]. Other models position co‐production as an overarching methodology that involves patients and the public throughout an entire project, from priority setting to implementing and evaluating the outcome [19]. Under this model, co‐design sits as the ‘solution developing’ phase of co‐production [19]. We chose to adopt a co‐design approach based on its congruence with our aim to develop a complex intervention [15].

Co‐design has been used to develop interventions in other populations including head and neck cancers and critical care survivors [20, 21]. Co‐design approaches have also been successfully utilised in operable and non‐operable lung cancer populations, predominantly targeted at screening, palliative care, and improving overall patient experience [22, 23, 24, 25]. Whilst qualitative studies report patients with operable and inoperable lung cancer's needs and preferences regarding exercise/rehabilitation programme design, to our knowledge none have adopted a co‐design approach [26, 27]. Therefore, this study aimed to use co‐design methodology to understand the key design requirements of an exercise‐based intervention to support people with operable lung cancer to prepare for and recover from surgery.

## 2 Methods

### 2.1 Study Design

This study was designed using the principles of experience‐based co‐design (EBCD) [28]. This utilises the knowledge and lived experiences of participants as the starting point for intervention design, ensuring that the goals and outcomes of the project are reflective of the needs and preferences of end‐users [28, 29]. Full details of the methodology, including a reflexivity statement, are provided in Supporting Information: File 1. The Consolidated Criteria for Reporting Qualitative Studies (COREQ) [30] and the Guidance for Reporting Involvement of Patients and The Public 2 (GRIPP2) [31] (Supporting Information: Tables 1 and 2) checklists were utilised.

Local institutional ethics approval was obtained before study commencement, with all participants providing informed written or verbal consent.

### 2.2 Sampling

Participants were recruited into two groups. Group 1 consisted of patients and their caregivers. Group 2 consisted of multidisciplinary professionals working in lung‐cancer‐related fields (e.g., physicians, surgeons, nurses, allied health professionals, advocates and researchers). Eligibility criteria and recruitment strategy for each group are provided in Table 1. The target sample size for each group was 5–15 participants based on The Point of Care Foundation guidelines for conducting EBCD [21, 32].

**Table 1:** Inclusion and exclusion criteria for participant groups.

	Inclusion criteria	Exclusion criteria	Method of approach
Group 1: Patients and caregivers	Adults aged over 18 with a diagnosis of confirmed lung cancer who had undergone thoracic surgery, and caregivers (did not have to participate as a patient‐carer dyad) Able to participate in a workshop conducted in English	Pre‐existing documented cognitive impairment limiting the ability to participate Caregivers of deceased individuals	Invitation mailed to purposively sampled list of patients and caregivers generated from the research team's database Advertised via consumer newsletters and conferences
Group 2: Professionals	Actively working healthcare professionals with prior experience providing care to patients with lung cancer in any setting (e.g., hospital and/or community settings) who have undergone thoracic surgery Or actively working employees of relevant organisations involved in the provision/development of advocacy, research, education and/or policy/legislation in lung cancer (e.g., educational institutions, Lung Foundation Australia, etc.).	None	Invitation emailed to interested clinicians from research team's database Advertised via social media, conferences and organisation newsletters Where key gaps were identified (e.g., key disciplines were underrepresented), local professionals, who were contacts of the research team, were invited directly via email

### 2.3 Data Collection

Demographic data were collected for all participants. The Capability, Opportunity, Motivation and Behaviour model (COM‐B) and Theoretical Domains Framework (TDF) underpinned ongoing qualitative data collection methods [33, 34], which involved a three‐stage approach [21, 32, 35]. Semi‐structured guides were developed/pilot tested within the research team and used in all stages:

1. Individual semi‐structured interviews: A sub‐group of at least three participants from each group were purposively sampled based on gender (Group 1) and discipline (Group 2) and invited to participate in video interviews. Consenting individuals were interviewed and video recorded. Footage was condensed into a 10‐min ‘trigger film’ to be played at the commencement of each Round 1 workshop. Participants were given the opportunity to review the footage and request edits.
2. Round 1: Separate 2‐h workshops for each group. Throughout the discussion, participants were guided to explore the touchpoints across the care continuum using an emotional mapping approach.
3. Round 2: Joint 2‐h workshops to explore Round 1 findings, service gaps and priorities, and to collaborate in the continued identification of the key intervention design requirements.

EBCD methodology uses separate workshops before joint co‐design workshops to enhance patient and caregiver comfortability, confidence to talk openly, and freedom to express honest thoughts and preferences before combining with the professionals [32]. Participants who could not participate in workshops were invited to participate in individual semi‐structured interviews. All sessions were audio recorded, transcribed verbatim, and cross‐checked by a second independent researcher.

Sequential exploratory quantitative data collection were carried out using bespoke surveys to triangulate and build upon the generalisability and reliability of the qualitative data [35, 36, 37]. Surveys were developed and finalised before commencing each round of workshops. They explored participant satisfaction with the EBCD process, experiences regarding exercise/rehabilitation, and recommendations/preferences for the programme prototype using Likert scales and open‐text responses. The survey instruments are available on request.

### 2.4 Setting

Sessions were held using video conferencing software (Zoom) or in‐person pending participant preferences.

### 2.5 Data Analysis

Demographic and survey data were coded and exported into SPSS Version 29 for descriptive statistical analysis [38]. Transcribed interviews/workshops and open‐text survey data were imported into NVivo 14 [39] and analysed inductively using the steps of thematic analysis by two independent researchers [40]. Qualitative and quantitative findings were integrated [41]. Barriers and facilitators to participation in exercise/rehabilitation and the proposed intervention components were deductively identified from the data and mapped to their corresponding Behaviour Change Wheel (BCW) ‘Sources of Behaviour’ (aided by the COM‐B and TDF) and ‘Intervention Functions’, respectively [42]. The identified BCW intervention functions were then compared and mapped to the corresponding sources of behaviour to ensure they represented theory‐based interventions ‘*likely to be effective in bringing about that change*’ [42]. These findings were explored in more depth during round two to produce a final intervention prototype, reported using the Template for Intervention Description and Replication (TIDieR) checklist [43]. The intervention development process is reported as per the Guidance for reporting intervention development studies in health research guideline (GUIDED) (Supporting Information: Table 3) [44].

## 3 Results

### 3.1 Participant Recruitment, Flow and Demographics

**Figure 1:**
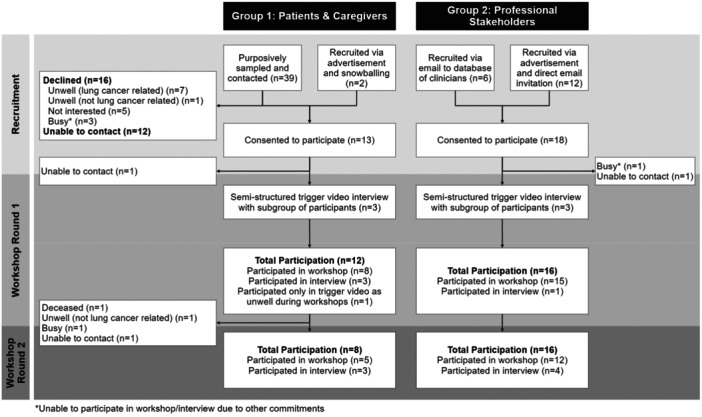
Flow of participants.

Thirty‐one participants (12 patients, one caregiver and 18 professionals) consented. Twenty‐eight participants (90% [11 patients, one caregiver, and 16 professionals]) participated in the first round of workshops/interviews, and 24 (77% [7 patients, one caregiver and 16 professionals]) participated in the second round (Figure 1). The retention rate between workshop rounds was 86% (24/28). Six workshops (*n* = 2–6) were held in Round 1, and three (*n* = 5–6) in Round 2 (Supporting Information: File 1). Participant demographics are summarised in Table 2. Post‐workshop survey findings (experience and opinion surveys) are summarised in Supporting Information: File 2.

**Table 2:** Demographics of participants.

Demographics^A^	Group 1 (*n* = 12), *n* (%)	Group 2 (*n* = 16), *n* (%)
Stakeholder group		
Patient	11 (92)	0
Caregiver	1 (8)	0
Professional	0	16 (100)
Age categorised in years		
25–34	0	7 (44)
35–44	0	5 (31)
45–54	1 (8)	3 (19)
55–64	3 (25)	1 (6)
65–74	5 (42)	0
75–84	3 (25)	0
Female	7 (58)	14 (88)
Highest level of education		
Some/completed primary school	1 (8)	0
Some/completed secondary school	4 (33)	0
Some/completed trade school/TAFE	4 (33)	0
University degree	1 (8)	12 (75)
Coursework post‐graduate studies	2 (17)	1 (6)
Research master's or doctorate	0	3 (19)
Geographical setting^B^		
Metropolitan		
Inner Metropolitan	2 (17)	14 (87.5)
Outer Metropolitan	6 (50)	1 (6)
Regional		
Inner Regional	3 (33)	1 (6)
Ethnicity		
White	10 (83)	
Asian	2 (17)	
Language spoken at home		
English	10 (83)	
English and other^C^	2 (17)	
Employment status		
Retired	7 (58)	
Employed (full or part‐time)	2 (17)	
Other^D^	3 (25)	
Time since first lung cancer surgery, years, median [IQR]	1 [1–2]	
NSCLC diagnosis *n*, (%)	11 (100)	
Lung cancer stage at diagnosis		
I	6 (55)	
II	2 (18)	
III	2 (18)	
IV	1 (9)	
Surgical approach		
Thoracotomy	2 (18)	
VATS/RATS	9 (82)	
Type of lung resection		
Lobectomy	8 (73)	
Segmentectomy	1 (9)	
Wedge resection	2 (18)	
Hospital length of stay (*n* = 10), days, median [IQR]	3.5 [2.75–9]	
Neoadjuvant treatment		
None	10 (91)	
Chemotherapy and Radiotherapy	1 (9)	
Adjuvant treatment		
None	8 (73)	
Chemotherapy and Radiotherapy	1 (9)	
Radiotherapy and Targeted Therapy	1 (9)	
Targeted Therapy	1 (9)	
Participated in exercise programme post‐operatively		
Yes	7 (64)	
Professional Discipline		
Physiotherapist		9 (56.3)
Nurse		2 (13)
Respiratory physician		2 (13)
Thoracic surgeon		1 (6)
Researcher		1 (6)
Peer support coordinator		1 (6)
Primary work setting		
Clinical		
Surgery		1 (6)
Inpatients		4 (25)
Outpatients (pre and/or post‐operative)		4 (25)
Across all settings (inpatients and outpatients)		3 (19)
Research		2 (13)
Community or charitable organisation		2 (13)
Service funding (clinical settings) (*n* = 12)		
Public		9 (75)
Private		3 (25)

Abbreviations: IQR, interquartile range; NSCLC, non‐small cell lung cancer; RATS, robotic‐assisted thoracic surgery; VATS, video‐assisted thoracic surgery.

^A^ At time of first encounter (excludes participants who consented but did not participate [Figure 1]).

^B^ Home location (Group 1) or workplace location (Group 2) [45].

^C^ Filipino (*n* = 1) and Mandarin and other dialects (*n* = 1).

^D^ Sick leave (*n* = 1), volunteer (*n* = 1) and full‐time caregiver (*n* = 1).

### 3.2 Workshop Round 1 – Themes

**Figure 2:**
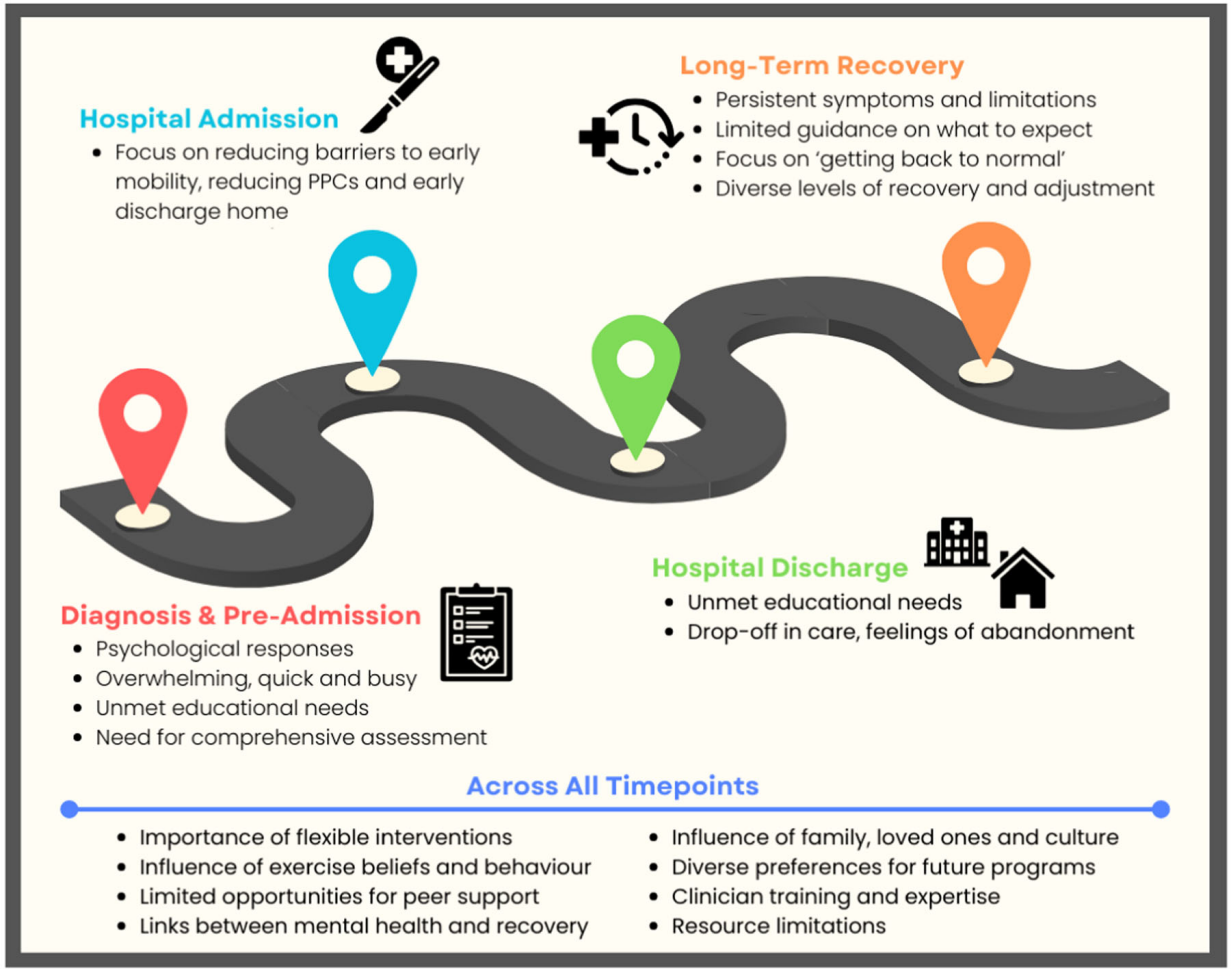
Summary of major themes from semi‐structured trigger video interviews and workshop Round 1. This figure provides an overview of the major themes from Round 1, mapped to the timepoints explored within the workshops across the pathway of operable lung cancer care.

Main themes are summarised below and in Figure 2 and Table 3. Sub‐themes and additional supporting quotes are provided in Supporting Information: Table 4.

**Table 3:** Major themes from trigger video interviews and workshop Round 1.

Diagnosis and preadmission	
Theme 1	Diverse emotional and psychological responses to diagnosis
Theme 2	The time between diagnosis and surgery is fast‐paced and overwhelming
Theme 3	Patients have unmet educational needs before surgery
Theme 4	Need for comprehensive pre‐operative assessment

Inpatient hospital admission	
Theme 5	Inpatient rehabilitation focuses on respiratory optimisation and facilitating timely hospital discharge

Hospital discharge	
Theme 6	Need for additional education to support hospital discharge and recovery
Theme 7	Perceived drop‐off in care and support post‐discharge can lead to feelings of abandonment

Long‐term recovery	
Theme 8	Experiences of persistent symptoms and difficulty returning to ‘normal’
Theme 9	A lack of education leads to difficulty managing symptoms and contextualising recovery
Theme 10	Recovery should focus on patients' goals and ‘getting back to normal’
Theme 11	Diverse levels of recovery and acceptance/adjustment

Across all timepoints	
Theme 12	Importance of flexibility and individualisation of programmes
Theme 13	Influence of pre‐existing habits and beliefs surrounding exercise
Theme 14	Lack of opportunities for peer support
Theme 15	The inextricable links between mental health, mindset and recovery
Theme 16	The influence of family/loved ones and culture
Theme 17	Preferences for optimal programme design differ, but continuity and follow‐up should be emphasised
Theme 18	Clinician training and upskilling
Theme 19	Limited resources and established models of care are current barriers

#### 3.2.1 Diagnosis and Pre‐Admission

The busyness of this period was seen as a barrier to pre‐operative education and prehabilitation. Some felt positively about the fast pace of this period and were grateful to have proceeded to surgery quickly.

“It just happened so quick with me though, I didn't really have time to think even about it…it was always moving, there was always some new appointment to go to.”

– Patient Workshop #1

Some participants reported unmet educational needs, and a desire for more specific, credible information. The importance of assessing individual patients' educational needs were highlighted.

“I got a leaflet…that sort of explained it, but yeah not really… I got told not to Google… but I mean, of course you do… I did most of it [sourcing education] myself.”

– Patient Trigger Video Interview #1

Participants felt that while some patients are told to exercise before surgery, more specific education, training (particularly skill‐building), enablement and incentivisation are required.

“When people have got these symptoms, it's knowing how much they can push themselves and not feel like they're risking making things worse, so the confidence to remain active and not worry.”

– Professional Workshop #1

Professionals felt that a more comprehensive, multi‐modal pre‐operative assessment is required to develop a more thorough understanding of patients' needs.

“I think like a comprehensive assessment as well…so that we can identify any other symptoms or any other needs that the patient has and then like an individualised programme.”

– Professional Workshop #3

#### 3.2.2 Inpatient Hospital Admission

Participants recalled facing barriers post‐operatively including unexpected pain, breathlessness, nausea, insomnia, and feelings of reduced autonomy.

“The first time I went to stand up, under supervision, I thought I was going to pass out. It was such a massive effort.”

– Patient Trigger Video Interview #3

#### 3.2.3 Hospital Discharge

Participants reported unmet educational needs at hospital discharge, particularly regarding guidance for family, medication management, and rehabilitation.

“It's something that your family's not trained for either, they don't expect it……”

– Patient Workshop #1

Professionals viewed pain, nausea and pain relief side‐effects as barriers to providing effective post‐operative education.

“…it's incredibly hard to be receptive to receiving education and information if you're in a lot of pain.”

– Professional Workshop #3

Feelings of abandonment and a drop‐off in support after hospital discharge were commonly reported, accompanied by feelings of isolation and a desire for a more formalised follow‐up system that emphasises continuity.

“Anyway, so they were great, they were right there with me and checking me the whole time [in the hospital]…. But soon as I was discharged it was like I was invisible.”

– Patient Workshop #2

Professionals viewed the current model of post‐discharge care as inadequate, reactive, and unstructured.

#### 3.2.4 Long‐Term Recovery

Persistent symptoms such as fatigue, breathlessness, and pain influenced participants' recovery and ability to return to their ‘normal lives’.

“After the surgery… I got easily tired… then I could feel a sort of shortness of breath.”

– Patient Workshop #3

This was particularly prominent among those having neoadjuvant and adjuvant therapies.

“And then all of a sudden, I was like, yeah, I couldn't walk across the road… I couldn't go for a walk around the block. No way.”

– Patient Interview #3

Some participants reported minimal ongoing symptoms. Others often reported feelings of frustration accompanied by a sense of being ‘failed’ by their body.

“See I'm a bricklayer concreter by trade, so I've lost a lot of energy, I know that, a lot of strength, I can't lift the things I used to be able to lift.”

– Patient Interview #1

Many participants felt persistent symptoms came as a surprise and reported feeling left in the dark regarding potential symptoms and their management, and how a ‘normal’ recovery might look.

“It's really quite frightening, um, to be coughing and feeling a lot of pain and there…there really didn't seem to be anybody that I could easily talk to…is this a problem? Should I be worried…is this normal?”

– Patient Trigger Video Interview #3

Participants felt that recovery and rehabilitation should focus on patient goals and facilitating a return to ‘normal life’, that is, promoting independence, self‐management and return to work and other meaningful activities.

“You know, ultimately, whatever we do, whatever cohort of patients we're dealing with, we want to give them the best life that they can be living.”

– Professional Workshop #2

#### 3.2.5 Across All Timepoints

Participants emphasised the importance of flexibility and individualisation, and the need for equity of care, particularly across geographical locations, and digital and health literacy.

“Yeah, some people are computer illiterate so – I'm pretty hopeless with computers as far as looking up stuff even if I'm interested.”

– Patient Interview #1

“Just considering like we're also rural/remote down here and travel is a big thing, cost is a big thing and also health literacy is a huge component as well.”

– Professional Workshop #1

Pre‐existing exercise habits, and positive views of exercise, were seen as key facilitators. Conversely, some participants viewed themselves as someone who did not exercise or did not view exercise as an important component in recovery.

“Look, I can't really imagine how a pre‐surgery exercise would help. I just can't see how that would help with the cancer.”

– Patient Interview #2

Some participants reported an unmet desire to hear from other patients with lived experience to build an understanding of the road ahead.

“If I could have gone to a website where there was just people's ‐ just their accounts of their life and how it affected them and maybe even videos of it if I didn't want to sit there reading it, anything to do with the people.”

– Patient Workshop #1

Participants reported an inextricable link between mental health, mindset and recovery, felt unprepared for the influence recovery may have on their mental health, and agreed the current model of care does not meet patients' mental health needs.

“The mental health side of it's half the battle. You get the mental health side right then the physical stuff follows suit.”

– Patient Workshop #2

Intervention preferences were diverse, but key preferred features of a future programme included formalised follow‐up, a multi‐modal approach, and continuity. Patients preferred home‐based interventions where possible.

Professionals suggested pragmatically utilising available programmes/resources and highlighted barriers to implementing ‘gold‐standard’ care including service availability, waitlists, staffing and funding.

A summary of the findings from participant subgroups is provided in Supporting Information: File 3.

Barriers, facilitators, and proposed intervention functions raised in the first round of workshops are summarised in Table 4, with further detail provided in Supporting Information: Tables 5–7. These were then further explored during Round 2 while refining the intervention prototype.

**Table 4:** Deductively identified barriers and facilitators to exercise/rehabilitation and intervention functions mapped to the Behaviour Change Wheel (BCW) [42].

COM‐B Components		TDF Domains	Current Barriers^A^	Current Facilitators^A^	BCW Intervention Functions^B^
Capability	Psychological	Knowledge	Lack of knowledge re:	Clinician‐delivered education and pamphlets	Education
			‐ Potential symptoms and their management	Access to pre‐existing materials online	
			‐ ‘Normal’ vs ‘not normal’ recovery	Clinician knowledge/confidence	
			‐ Importance/rationale of exercise		
			‐ Diagnosis, implications, and management		
			Lack of awareness re:		
			‐ Available avenues for support and follow‐up		
			‐ Evidence‐based practice (clinicians)		
		Skills (cognitive and interpersonal)	Lower digital literacy	Adequate digital literacy	Training
			Lower health literacy	Adequate health literacy	
			Limited family member capability to provide ‘caregiver’ role		
			Knowledge of referral processes (clinicians)		
		Memory, attention and decision processes	Overwhelming nature of pre‐operative period	View of self as someone who needs to be informed to cope	Training
			Lack of resources to refer to at own pace		Environmental restructuring
			No desire for education – ‘the less I know, the better’		Enablement
		Behaviour regulation	Pre‐existing sedentary behaviour	Pre‐existing active behaviours	Training
			Limited ability to monitor own symptoms and recovery		Modelling
			Lack of support to understand/break ‘unhelpful’ habits		Enablement
	Physical	Skills	Reduced exercise tolerance/fitness	Pre‐morbidly high exercise tolerance, fitness, and muscle strength	Training
			Persistent symptoms e.g., pain, fatigue, breathlessness	Absence of or few symptoms	
			Treatment side‐effects		
Opportunity	Physical	Environmental context and resources	Isolation/poor access to support due to geographical location	Implicit trust/gratitude in care team	Environmental restructuring
			Limited flexibility of existing services	Utilisation of reimbursed/publicly funded programs	Enablement
			Financial cost of exercise programs	Utilisation of existing services	
			Unavailability of exercise programs	Metropolitan geographical location	
			Long waitlists		
			Lack of healthcare resources		
			Goals/priorities of existing clinical pathways		
			Differing service availability between regions/funding models		
	Social	Social influences	Lack of clinician continuity	Existing opportunities for peer support	Environmental restructuring
			Competing priorities e.g., caregiving and work	Family/loved one's encouragement	Modelling
			Stigma	Family/loved ones offloading patients' responsibilities	Enablement
			Family/cultural beliefs around rest		
			Loved ones' limited understanding of symptoms		
			Lack of opportunities to connect with others with lung cancer		
Motivation	Reflective	Beliefs about capabilities	Lack of self‐efficacy, determination or grit	High self‐efficacy, determination and grit	Education
			Inability to contextualise own recovery/progress	Belief that it is possible to exercise despite barriers	Persuasion
			Grief and adjustment to symptoms/impairments		Modelling
			Belief that ‘I do not need rehabilitation’		
			Reduction in self‐perceived ability		
		Social/professional role	View of self as ‘inactive/lazy’	View of self as ‘active’	Education
			Lack of clinical oversight/responsibility for referrals		Enablement
		Beliefs about consequences	Declining support due to limited understanding of symptoms	Belief that exercise/rehabilitation is important for recovery	Education
			Belief that structured exercise confers no benefit over incidental activity	Belief that exercise is important for general health	Persuasion
					Modelling
		Optimism	Negative view of likely prognosis/outcomes	An ‘operable’ diagnosis	Education
			View that it is too late to exercise after diagnosis/before surgery		Enablement
			Belief that likely outcomes cannot be augmented		
		Intentions	Limited oversight of rehabilitation/recovery	Decision to incorporate exercise into daily routine	Education
			Reduced motivation		Persuasion
					Incentivisation
					Modelling
		Goals	Lack of support to set and achieve goals	Goals to return to previously active lifestyle	Persuasion
			Low prioritisation of exercise/recovery	Belief that exercise will help ‘fight’ against cancer	Incentivisation
					Modelling
					Enablement
	Automatic	Emotions	Feeling deserted/isolated from health system	Maintaining a positive mindset	Persuasion
			Worry, anxiety, fear for the future		Modelling
			Lack of enjoyment of exercise		Enablement
		Reinforcement	Reliance on encouragement from others	Benefit of encouragement from others	Training
			Exacerbating symptoms during exercise reinforcing sedentary behaviour	Feeling better/reduced symptoms after exercising	Incentivisation
					Environmental restructuring

*Note:*

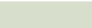
 = Whole cohort voiced.

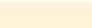
 = Group 1 (patients/caregivers) voiced.

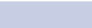
 = Group 2 (professionals) voiced.

A Deductively identified barriers and facilitators to exercise/rehabilitation participation mapped to relevant COM‐B components and TDF domains.

B Deductively identified BCW intervention functions proposed by participants mapped to their corresponding COM‐B components and TDF domains [42]. The proposed intervention functions are associated with each TDF domain and not individual barriers and facilitators. The proposed intervention components are provided in Supplementary Table 7.

Abbreviations: BCW, Behaviour Change Wheel; COM‐B, Capability, Opportunity, Motivation – Behaviour Model; TDF, Theoretical Domains Framework.

There were varying degrees of convergence and divergence between the qualitative and quantitative findings. A summary of meta‐inferences and interpretations is provided in Supporting Information: Table 8.

### 3.3 Workshop Round 2 – Intervention Prototype

**Figure 3:**
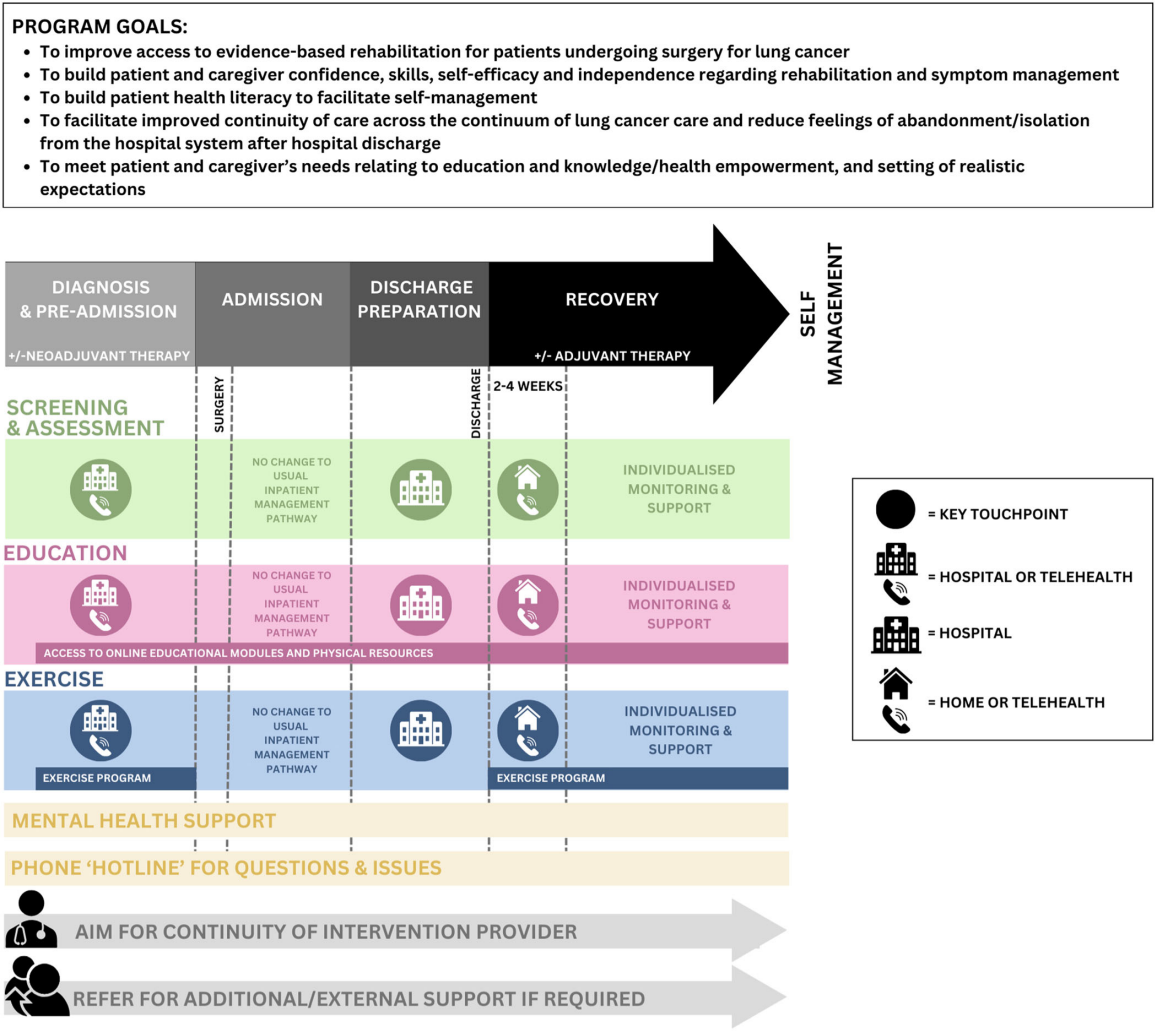
Visual overview of the proposed intervention prototype. The figure demonstrates the main components of the intervention prototype (screening and assessment [green], education [pink] and exercise [blue]), and the key timepoints of delivery (diagnosis/pre‐admission, discharge preparation and recovery). Dotted lines indicate key events and timelines relevant to the intervention. Important adjuncts such as mental health support and a phone ‘hotline’ (yellow) are additional key intervention requirements, and important principles such as continuity and referral to external support if required (grey) underpin the intervention across the continuum.

Figure 3 provides a high‐level summary and Supporting Information: Table 9 provides a more detailed proposal of the co‐designed intervention prototype. The main BCW intervention functions proposed to target the identified barriers and facilitators were enablement, environmental restructuring, training and education (Supporting Information: Table 7).

#### 3.3.1 Enablement, Environmental Restructuring and Training

Participants proposed a flexible, individualised and multimodal model of rehabilitation commencing as close to diagnosis as possible. Initial screening and education were preferred to take place with a clinician in person or via telehealth. Most participants agreed that an independent, self‐paced exercise programme was most feasible pre‐operatively, accompanied by specific education and enablement regarding the importance of pre‐operative exercise, how to exercise effectively, and ways to self‐monitor safety and technique.

No changes were proposed to the standard inpatient management pathway, which typically includes early mobilisation, deep breathing exercises, and upper limb/thoracic spine mobility exercises [8]. A checklist‐supported ‘check‐in’ was suggested to support patients 24–48 h before hospital discharge, designed to screen for symptoms, impairments and activity limitations; progress so far; any ongoing education gaps; future support needs; and generate a plan for post‐discharge exercise and follow‐up.

Most gaps in the current model were identified in the post‐operative long‐term recovery time point. The proposed model of follow‐up, incorporating an initial early check‐in following hospital discharge (within two to four weeks), was designed in response to patients' perceptions of abandonment in this period. Patients preferred, where possible, for this initial appointment to occur via a home visit, however, professionals expressed concerns about the feasibility of this and felt telehealth could serve as an adequate replacement where appropriate. The prototype model provides varying levels of support based on needs and preferences underpinned by a strong education and long‐term habit formation/self‐management approach.

The workshop guide did not focus on eliciting specific preferences regarding the optimal intervention provider, in an attempt to ensure the developed prototype was pragmatic and implementable across different settings and healthcare staffing models. However, it was generally suggested by participants that clinicians with backgrounds in physiotherapy or nursing may be appropriate providers. The prevailing critical component regarding the provider was continuity, aiming for a sole clinician to provide the entire intervention where feasible and appropriate.

Mental health support was highlighted as a key intervention component, commencing at intake and continuing until programme discharge. Due to barriers accessing psychological services, participants felt that this could be provided by the primary intervention provider (nurse or physiotherapist), with a focus on normalising mental health and improving self‐management skills (e.g., cognitive behavioural approaches, meditation and mindfulness), self‐efficacy, and confidence. Clinician upskilling was proposed to facilitate continuity and connection via utilising one primary provider. Professionals also emphasised the importance of escalating patients with clinical levels of distress to appropriate psychological services and/or back to primary care physicians.

#### 3.3.2 Education

A flexible, individualised approach to education was proposed, given the variability of patients' needs and preferences, incorporating an assessment of patients' educational needs. Commonly desired education topics included further detail on the diagnosis, what to expect while in hospital, symptoms and their management, exercise and past patients' experiences. It was proposed that the bulk of the education should commence with a clinician and occur as close to diagnosis as possible and be reiterated at multiple time points. While some patients preferred an online, self‐paced education module, many reported low levels of digital access and literacy, highlighting the need for physical materials, and the integration of digital skill‐building strategies.

## 4 Discussion

The EBCD process identified key gaps in the current operable lung cancer model of care and key intervention functions targeted at overcoming these gaps. By facilitating the implementation of exercise‐based rehabilitation, this patient‐centred intervention prototype has the potential to improve the quality of operable lung cancer care, patient experience, and patient and health‐system outcomes, for example, by supporting the routine delivery of pre‐operative exercise, which can reduce both the risk of post‐operative pulmonary complications and hospital length of stay [3].

The co‐designed intervention prototype represents a novel approach compared to routine practice. In Australia, where this co‐design study was conducted, the most common programmes servicing this population are ‘pulmonary rehabilitation’ and ‘oncology rehabilitation’ [8]. Our recent Australian survey explored the availability, delivery, and content of these programmes, all of which differed significantly between health services [8]. Most pre‐ and post‐operative programmes provided centre‐based, supervised exercise training programmes and most post‐operative programmes were delivered in a group‐based format [8]. While some post‐operative programmes offered supervised telehealth sessions as an option, few provided in‐person, home‐based sessions [8]. No pre‐operative and only four post‐operative services were identified in regional or rural areas [8]. Patient and caregiver co‐design participants typically were not interested in participating in structured, centre and/or group‐based programmes. As such, our prototype is predominantly conducted in the home environment, where appropriate patients participate in independent pre‐ and post‐operative exercise, supported by initial face‐to‐face (home or centre‐based) or telehealth sessions with an exercise provider. We propose a scaled approach whereby patients are monitored at a length and frequency based on their individual needs, and those identified as needing additional support through screening are referred to these more traditional supervised, centre‐based programmes.

Currently, screening and assessment, particularly in the pre‐operative setting, are typically ad hoc, and few services comprehensively screen the domains we have proposed within our prototype, such as mobility, physical activity levels, physical function, self‐efficacy, symptoms, etc (Supporting Information: Table 9) [8]. The proposed prototype provides an overview of the key domains to screen and assess at different timepoints from the perspectives of patients, caregivers, and professionals. Education and exercise prescription also appear to vary from service to service [8]. For example, in our survey only 50% of pre‐operative programmes surveyed provided education on lung cancer exercise guidelines to at least ‘some’ patients, and 50% prescribed a home‐based exercise programme to ‘all’ patients [8]. Most post‐operative outpatient services appeared to prescribe home exercise programmes and educate most patients about behaviour change, symptom self‐management, and lung cancer exercise guidelines [8]. Our prototype recommends a front‐ended education approach, where exercise and self‐management education commences pre‐operatively and is reinforced at specified timepoints throughout lung cancer treatment. Proposed programme adjuncts such as embedded mental health support and the ‘hotline’ are also novel compared to usual practice. Our prototype's emphasis on continuity also appears to be novel compared to current practice, as even at health services where pre‐ and post‐operative exercise was available, most respondents only worked with patients at one timepoint [8].

### 4.1 Addressing Implementation Barriers

The co‐designed intervention prototype addresses implementation barriers by providing an alternative to traditional rehabilitation programmes, which typically have long waitlists and are scarcely available in regional and remote areas [8]. Emerging evidence supports the efficacy of home‐based and telehealth lung cancer exercise programmes [46, 47]. However, issues of adherence, resources, and digital and health literacy hamper their implementation [47]. Our proposed intervention contains inbuilt strategies to overcome these barriers.

Identified key barriers to exercise participation included persistent symptoms and fear of symptom exacerbation. These barriers have also been previously identified in the literature [48, 49, 50]. Additional findings from the present study include the importance of utilising patient‐preferred terminology to further support exercise enablement, that is, participants tended to agree that ‘fitness’ may have more positive connotations than ‘exercise’, given potential pre‐existing beliefs around exercise, or past experiences of symptom exacerbation during clinical exercise tests as part of surgical workup. These findings highlight the importance of co‐designing education materials, emphasising and front‐ending education relating to symptom management and exercise safety, and ensuring education is readily available in flexible and accessible formats.

Clinician knowledge and application of exercise research is another barrier our prototype seeks to address, with prior research showing that current operable lung cancer practice is influenced significantly by clinicians' personal experience [6, 8]. This further supports the need for a targeted approach to clinician education and upskilling, focused on improving clinician knowledge of exercise guidelines and competency to provide the intervention.

The prototype recommends utilising a single primary intervention provider to facilitate continuity, trust and connection, acknowledging that the chosen provider would require targeted upskilling. For example, a nursing provider may require upskilling in exercise prescription, and a physiotherapist provider may require wound and medication management upskilling. Additionally, the provider would require training in cognitive behavioural approaches to self‐management, as has been suggested and utilised in other respiratory and cancer populations [51, 52].

### 4.2 Embedding Principles of Health and Digital Literacy

Health and digital literacy were raised as barriers by both participant groups, and as such, it was vital to embed considerations and support within the prototype. Health literacy is a key factor in successful disease management and has been correlated with health outcomes in other conditions [53]. Low rates of health literacy are common among patients with lung cancer [54, 55]. Given the proposed intervention aims to provide individualised patient education and facilitate self‐management, incorporating health literacy assessment and a responsive approach to enablement and skill development for patients with lower health literacy is essential. The proposed prototype and implementation strategy incorporate suggested strategies for developing health literacy‐responsive interventions, including assessing health literacy and learning needs, ensuring adequate provider training, utilising virtual communication strategies, and partnering with stakeholders/consumers [53, 56].

There is increasing interest in digital health interventions for patients with lung cancer [57]. However, as in our participant group, previous research has identified large sub‐groups of patients with lung cancer with inadequate digital access and/or literacy to participate [58, 59, 60]. Lower education levels have been correlated with lower digital literacy in patients with lung cancer [60]. Older age, regional/remote geographical setting, and reduced social support have been associated with lower digital literacy among a broader cancer cohort [61]. To provide equitable rehabilitation, our prototype incorporates suggested strategies, including collaborating with end‐users to ensure content and layouts are basic and culturally responsive, embedding and assessing accessibility, incorporating opportunities for digital skill building, and ensuring non‐digital services remain available [62, 63, 64].

### 4.3 Strengths and Limitations

Our approach to co‐design resulted in high participant satisfaction and a high retention rate between both workshop rounds. We successfully recruited a diverse cohort of participants, further supporting the generalisability of our findings. The incorporation of stakeholder involvement and use of theoretical frameworks into the codesign process further strengthens the rigour of the prototype [65]. Some participants had previously participated in a remotely delivered exercise programme, most (*n* = 10/11) lived in the same state and received their lung cancer care at the same health service and therefore experienced similar care pathways, and several participants declined to participate due to severe symptom burden, all of which may have influenced our findings. Due to the small number of caregivers recruited and the exclusion of non‐English speaking participants, we cannot draw any meaningful conclusions about the needs of these groups. Despite mental health arising as a topic, we did not recruit any mental health professionals. While we did aim to facilitate the authentic involvement of patients and the public throughout data collection, they were not directly involved in project priority setting, planning and conducting the workshops, or data analysis.

### 4.4 Future Directions

Future lung cancer research should assess and investigate the influence of health and digital literacy on rehabilitation participation, and ways to build patients' digital self‐efficacy and skills. The most acceptable and feasible approaches to clinician upskilling should also be investigated. The future directions of this research programme include a multi‐stage approach to implementation, incorporating further co‐design and user testing of intervention materials, feasibility, acceptability and efficacy testing (including a process evaluation) and development of strategies for implementation, sustainability and scalability [66].

## 5 Conclusion

Using an EBCD process, participants collaborated to identify strategies and facilitators in recovery, culminating in the co‐design of the proposed multi‐modal intervention prototype, incorporating a flexible, individualised approach to pre‐ and rehabilitation.

## Author Contributions

**Georgina A. Whish‐Wilson:** conceptualization, methodology, investigation, formal analysis, visualization, writing–original draft, funding acquisition, writing–review and editing, project administration. **Lara Edbrooke:** conceptualization, methodology, investigation, validation, writing–review and editing, funding acquisition, supervision. **Vinicius Cavalheri:** methodology, writing–review and editing, funding acquisition. **Zoe T. Calulo Rivera:** methodology, investigation, writing–review and editing. **Madeline Cavallaro:** methodology, investigation, writing–review and editing. **Daniel R. Seller:** methodology, writing–review and editing, funding acquisition. **Catherine L. Granger:** conceptualization, methodology, investigation, validation, writing–review and editing, funding acquisition, supervision. **Selina M. Parry:** conceptualization, methodology, investigation, formal analysis, writing–review and editing, funding acquisition, supervision.

## Ethics Statement

Local institutional ethics approval was obtained (ID: 23070).

## Conflicts of Interest

SMP is currently a recipient of the Al and Val Rossenstrauss Fellowship. LE is currently a recipient of a Victorian Cancer Agency Fellowship. Funders had no input into the design, implementation, or analysis of this study. The other authors declare no conflicts of interest.

## Supporting information

Supporting information.

## Data Availability

The data that support the findings of this study are available from the corresponding author upon reasonable request.
