# Supplementary material for: Empowering Recovery: A Co‐Designed Intervention to Transform Care for Operable Lung Cancer
Source: Health Expect. 2025 Apr 5;28(2):e70196. doi: 10.1111/hex.70196 (PMC11971569; doi:10.1111/hex.70196)
Supplement: Supplementary file 1 — Supporting information. [file HEX-28-e70196-s001.docx]

**Supplementary File 1: Methods and Reflexivity**

*Research team and reflexivity statement (Consolidated criteria for reporting qualitative research [COREQ] Domain 1)*

The research team was comprised of physiotherapist clinicians and clinician-researchers, with interests in cancer, respiratory and critical care research. The primary workshop facilitators were GAWW and SMP.

The first author, GAWW (she/her, BPT AdvRes(Hons), PhD candidate in physiotherapy) had >3 years of experience in conducting and analysing qualitative research. She completed mentorship in qualitative research methods and EBCD with, and was supervised by, senior researcher SMP. Throughout the project, she was working clinically as a senior clinician physiotherapist in acute cancer care and had interests in cancer, exercise oncology, and cardiorespiratory physiotherapy.

SMP (she/her, PhD in Physiotherapy, Associate Professor) had > 10 years involvement in quantitative and qualitative health research with an interest in thoracic surgery and acute cardiorespiratory populations including those who are critically ill. SMP had formal training in qualitative and EBCD methodologies including training through The Point of Care Foundation.

GAWW conducted all semi-structured interviews and co-facilitated each workshop. A secondary facilitator was present in each session (SMP, CLG or LE) of whom all had experience in qualitative methodologies and delivering focus groups in online and in-person settings. Given GAWW was trained by SMP, there is potential that her positionality and approach to data collection and analysis were influenced by SMP.

SMP and GAWW independently analysed the first round of workshop data and collaborated to determine the themes. SMP and GAWW independently analysed the second round of workshop data and collaborated to map the intervention prototype. The final intervention prototype was cross-checked by LE and CG.

Due to their involvement in prior research conducted by our research team, some participants (10 out of 11 patients) had pre-existing relationships with the researchers (GAWW, CG and SMP) in a research capacity. However, it is important to note none had a current or prior clinical relationship with any team member in terms of receiving hospital or community care.

Additionally, our entire research team were from physiotherapy backgrounds, which likely ultimately influenced the overall direction, priorities and goals of the project. Participants were read a summary of the facilitators, their professional and clinical backgrounds, their research interests, assumptions and potential biases at the start of each interview and workshop. Workshop/interview guides, and session goals, were pre-determined to reduce the influence of individual facilitators.

*Study design and methods supplement (COREQ Domain 2)*

Participants were recruited in two groups: patients/caregivers (Group 1) and lung cancer professionals (Group 2). Group 1 were purposively sampled from the research team’s database of patients who underwent surgery in one major metropolitan public hospital that serviced a large catchment, including patients from regional and rural areas and interstate. No specific sampling strategy was used. However, care was taken to aim for diversity in gender, age, lung cancer stage, surgical approach, neo/adjuvant therapy and home location/rurality. We approached sampling pragmatically by first approaching a purposively sampled group of patients with as much diversity in these domains as possible and moving through this list to achieve our desired sample size. Advertisements were also made broadly via Australian and New Zealand consumer networks and interstate conferences to locate patients from different locations/health services. Group 2 were also recruited through the research team’s database of clinicians working in this area, including clinicians from every Australian state and territory and the North and South Islands of New Zealand. We also advertised broadly via social media, conferences, and organisations, and where required to achieve diversity among disciplines, we reached out to known local professionals directly.

The study was conducted in an Australian healthcare setting – a health system with both public (government-funded or subsidised) and private (private health insurer and self-funded) components. Most participants received care or worked within the public health system. This study was designed using the principles of EBCD, based on the Point of Care Foundation EBCD Toolkit and other previously published EBCD[1-3].Qualitative data collection was completed in a three-stage approach:

1. Individual semi-structured trigger video interviews: A sub-group of at least three participants from group one (patients and caregivers) and group two (lung cancer healthcare/research/advocacy) were purposively sampled based on gender (Group 1) gender and discipline (Group 2). This sub-group was interviewed using a semi-structured interview guide designed to guide participants to reflect on their experiences of the current operable lung cancer care pathway, with specific reference to exercise programs/support and rehabilitation. Interviews were conducted either in-person or via online video conference (Zoom) and were video recorded and condensed into a 10-minute compilation known as a ‘trigger film’ to be played at the commencement of each round one workshop[1]. The trigger film was edited by GAWW and reviewed/revised by SMP, CG and LE. The trigger film aimed to trigger memories, discussions, and conversations of workshop participants, and guide them to identify key moments, places, experiences, and emotions involved in their journey with lung cancer, known as ‘touchpoints’[1]. Participants of this stage were given the opportunity to review and approve the footage and request any edits prior to the film being finalised. No edits were requested. Three semi-structured trigger video interviews were conducted for each group (six interviews in total). Three of these were conducted online via Zoom (one Group 1 interview and two Group 2 interviews), and the rest were conducted in person. One participant from Group 1 who participated in a semi-structured trigger video interview became unwell and was unable to participate in the workshops.
2. Round One Workshops: Separate workshops were held for participant Group 1 and 2, commencing with the playing of the trigger film. The workshops were guided by a semi-structured workshop guide which encouraged participants to explore their own narratives and experiences navigating a lung cancer diagnosis and/or caring for somebody with lung cancer (group one) or providing lung cancer healthcare/advocacy/research (group two). Throughout the discussion, participants were guided to explore the touchpoints experienced along the pathway of operable lung cancer care using an emotional mapping approach, particularly relating to exercise and rehabilitation, as well as key service design requirements, and gaps, barriers, and facilitators of recovery.
3. Round Two Workshops: In the second round of workshops participants from both groups were brought together where possible. Workshops and interviews commenced by providing participants with a comprehensive overview of the themes and sub-themes, and the draft intervention prototype, generated from round one. Participants were then guided to jointly explore the findings, service gaps and priorities as identified during round one, and to collaborate in the continued identification of the key design requirements of the intervention prototype. Only participants who had participated in the first round of workshops were invited to participate in the second round.

In both workshop rounds, participants who were unable to participate (i.e., patients who were unable to join an online workshop due to digital literacy or access and were also unable to attend an in-person workshop due to travel distance or work commitments, and professional stakeholders who could not attend scheduled workshops due to work commitments) were invited to participate in individual semi-structured interviews using the same interview guide. The research team present during the workshops included: two co-facilitators, a field note-taker, and a technology-support delegate (online workshops only). No non-participants beyond the research team were present during data collection.

All workshops and interviews were guided by semi-structured interview guides, which were all pilot tested within the research team. All guides were underpinned by the COM-B (Capability, Opportunity, Motivation, Behaviour) model and the Theoretical Domains Framework (TDF)[4, 5]. All interviews and workshops were audio recorded, transcribed verbatim by a member of the research team or an external professional transcriber, and cross-checked by a second member of the research team for accuracy. GAWW either transcribed or cross-checked each transcript to ensure familiarity with the data. A research team member was present at each workshop to take field notes during each workshop.

Participants were encouraged to complete optional post-workshop/interview surveys, which asked for feedback on their experiences of the workshop/interview and served as an opportunity to provide additional information on topics that had been discussed. Each survey was pilot tested within the research team, and the content of the surveys was refined after piloting. Each survey took no longer than 15 minutes to complete and was intended to triangulate and build upon the generalisability and reliability of qualitative data obtained.

Rather than reaching data saturation, the focus was to enable all participants an opportunity to participate in both workshop rounds. Therefore, enough workshops and interviews were conducted to ensure all participants could participate in each round. Member checking of transcripts did not take place; however, participants were given the opportunity of supplying additional information via the post-workshop surveys.

*Analysis and findings (COREQ Domain 3)*

We purposefully over-recruited to group two (professionals) in an attempt to preserve the diversity of attendees with respect to discipline and gender due to the anticipated likelihood of dropouts, given that many were attending during a clinical workday with many competing priorities.

Each workshop ran for two hours. Semi-structured interviews across all three stages varied in length, ranging from 20-70 minutes. To maximise participation and overcome logistical barriers (e.g., participants from regional areas who could not travel to participate in person nor join online), six workshops were held in Round One (three workshops per group). The number of participants and mode of each workshop is provided in Supplementary Table 1.1. Two Group One (patient/caregiver) workshops in Round One only had two participants each due to the above logistical challenges and last-minute drop-outs due to illness.

**Supplementary Table 1.1: Workshop Participants and Mode**

| **Round 1** | | | |  |  |
| --- | --- | --- | --- | --- | --- |
|  |  | **Participants, n** | **Workshop Mode** | **Female,**  **n (%)** | **Regional geographic setting^A^,**  **n (%)** |
| **Group 1** | Workshop #1 | 2 | Online | 2 (100) | 0 |
|  | Workshop #2 | 2 | Online | 2 (100) | 2 (100) |
|  | Workshop #3 | 4 | In-Person | 2 (50) | 0 |
| **Group 2** | Workshop #1 | 6 | Online | 6 (100) | 1 (17) |
|  | Workshop #2 | 5 | Online | 4 (80) | 0 |
|  | Workshop #3 | 4 | Online | 4 (100) | 0 |
| **Round 2** | | | |  |  |
| **Mixed** | Workshop #1 | 6 | Online | 6 (100) | 2 (33) |
|  | Workshop #2 | 6 | Online | 5 (83) | 0 |
|  | Workshop #3 | 5 | Hybrid ^B^ | 4 (80) | 0 |
| ^A^ Home Location (Group 1) or Workplace Location (Group 2)[6]  ^B^ Group 1 participants attended in-person and Group 2 participants joined online. | | | | | |

Transcribed interview, workshop, and open-text survey data were imported into NVivo 14[7] and analysed qualitatively using the steps of thematic analysis by two independent researchers (GWW, SMP). Identified themes were organised according to the relevant timepoint or ‘touchpoint’ in the operable lung cancer care journey. Thematic analysis was an iterative process, completed after each of the three data collection steps as outlined above, and future semi-structured workshop/interview guides were based on the previous round(s) findings. Interviews were coded last and used to strengthen already formed themes to reduce the influence of any one participants’ voice. Throughout this process, data were routinely ‘queried’ based on participant demographics utilising inbuilt NVivo analysis functions to ensure patients' voices were highlighted, and that the themes and findings were representative of a broad range of views (i.e., not generated from the one workshop or participant voice). It was planned that, where possible, differences and/or patterns of responses from people with differing characteristics would be explored (e.g., gender, geographical location, cancer stage, neoadjuvant and/or adjuvant treatment, etc.) using the same inbuilt NVivo functions.

Barriers and facilitators to recovery were deductively identified throughout the analysis of data from all stages of qualitative data collection were mapped according to the Behaviour Change Wheel ‘Sources of Behaviour’, aided by the COM-B and TDF frameworks[8]. Proposed intervention components were mapped to the Behaviour Change Wheel ‘Intervention Functions’[8]. The identified BCW intervention functions were re-mapped to their corresponding sources of behaviour to ensure that they were aligned/congruent with previously published theory[8]. This allowed researchers to identify specific behaviours, barriers, and facilitators to address in the development of the intervention through the lens of behaviour change theory, facilitating the development of a highly specific intervention targeted to the participants’ needs. Proposed barriers, facilitators, and intervention functions were then explored in more detail during the third stage of data collection (workshop two) to produce a final intervention prototype, reported using the Template for Intervention Description and Replication (TIDieR) checklist[9].

Qualitative and quantitative findings were integrated during the ‘interpretation and reporting’ level of the study using the four steps suggested by Skamagki et al[10].

Member checking of the findings did not formally take place, however, the second round of workshops served as an avenue for participants to provide feedback on the findings from the first round including the intervention prototype.

**Supplementary Table 1: COREQ (COnsolidated criteria for REporting Qualitative research) Checklist [11]**

| **Topic** | **Description** | **Reported on Page No.** |
| --- | --- | --- |
| **Domain 1: Research team and reflexivity** | | |
| *Personal characteristics* | | |
| 1. Interviewer/facilitator | Which author/s conducted the interview or focus group? | Supplementary File 1, p.1 |
| 1. Credentials | What were the researcher’s credentials? E.g. PhD, MD | Supplementary File 1, p.1 |
| 1. Occupation | What was their occupation at the time of the study? | Supplementary File 1, p.1 |
| 1. Gender | Was the researcher male or female? | Supplementary File 1, p.1 |
| 1. Experience and training | What experience or training did the researcher have? | Supplementary File 1, p.1 |
| *Relationship with participants* | | |
| 1. Relationship established | Was a relationship established prior to study commencement? | Supplementary File 1, p.1 |
| 1. Participant knowledge of the interviewer | What did the participants know about the researcher? e.g. personal goals, reasons for doing the research | Supplementary File 1, p.1 |
| 1. Interviewer characteristics | What characteristics were reported about the interviewer/facilitator? e.g. Bias, assumptions, reasons and interests in the research topic | Supplementary File 1, p.1 |
| **Domain 2: Study design** | | |
| *Theoretical framework* | | |
| 1. Methodological orientation and theory | What methodological orientation was stated to underpin the study? e.g. grounded theory, discourse analysis, ethnography, phenomenology, content analysis | Main manuscript p.1 and Supplementary File 1 pp.2-3 |
| *Participant selection* | | |
| 1. Sampling | How were participants selected? e.g. purposive, convenience, consecutive, snowball | Main manuscript Table 1 |
| 1. Method of approach | How were participants approached? e.g. face-to-face, telephone, mail, email | Main manuscript Table 1 |
| 1. Sample size | How many participants were in the study? | Main manuscript p.3 |
| 1. Non-participation | How many people refused to participate or dropped out? Reasons? | Main manuscript Figure 1 |
| *Setting* | | |
| 1. Setting of data collection | Where was the data collected? e.g. home, clinic, workplace | Main manuscript p.4 and Supplementary File 1 pp.1-2 |
| 1. Presence of non-participants | Was anyone else present besides the participants and researchers? | Supplementary File 1 p.2 |
| 1. Description of the sample | What are the important characteristics of the sample? e.g. demographic data, date | Main manuscript Table 2 |
| *Data collection* | | |
| 1. Interview guide | Were questions, prompts, guides provided by the authors? Was it pilot-tested? | Main manuscript p.3 and Supplementary File 1 p.3.  Guides are available on request. |
| 1. Repeat interviews | Were repeat interviews carried out? If yes, how many? | N/A |
| 1. Audio/visual recording | Did the research use audio or visual recording to collect the data? | Main manuscript p.4 and Supplementary File 1 p.3 |
| 1. Field notes | Were field notes made during and/or after the interview or focus group? | Supplementary File 1 p.2 |
| 1. Duration | What was the duration of the interviews or focus group? | Supplementary File 1 p.3 |
| 1. Data saturation | Was data saturation discussed? | Supplementary File 1 p.3 |
| 1. Transcripts returned | Were transcripts returned to participants for comment and/or correction? | Supplementary File 1 p.3 |
| **Domain 3: Analysis and findings** | | |
| *Data analysis* | | |
| 1. Number of data coders | How many data coders coded the data? | Main manuscript p.4 and Supplementary File 1 p.3 |
| 1. Description of the coding tree | Did authors provide a description of the coding tree? | Available on request. |
| 1. Derivation of themes | Were themes identified in advance or derived from the data? | Main manuscript p.4 and Supplementary File 1 p.3 |
| 1. Software | What software, if applicable, was used to manage the data? | Main manuscript p.4 and Supplementary File 1 p.3 |
| 1. Participant checking | Did participants provide feedback on the findings? | Supplementary File 1 p.4 |
| *Reporting* | | |
| 1. Quotations presented | Were participant quotations presented to illustrate the themes/findings? Was each quotation identified? e.g. participant number | Main manuscript pp.5-7 and Supplementary Table 4 |
| 1. Data and findings consistent | Was there consistency between the data presented and the findings? | Main manuscript pp.4-8 and Supplementary Table 4 |
| 1. Clarity of major themes | Were major themes clearly presented in the findings? | Main manuscript Figure 2 and Table 3, and Supplementary Table 4 |
| 1. Clarity of minor themes | Is there a description of diverse cases or discussion of minor themes? | Supplementary Table 4 |

**Supplementary Table 2: GRIPP2 Guidance for Reporting Involvement of Patients and The Public 2 (GRIPP2) Checklist [12]**

| **Topic** | **Item** | **Reported on Page No.** |
| --- | --- | --- |
| **Section 1: Abstract of paper** | | |
| 1a: Aim | Report the aim of the study | Abstract |
| 1b: Methods | Describe the methods used by which patients and the public were involved | Abstract |
| 1c: Results | Report the impacts and outcomes of PPI in the study | Abstract |
| 1d: Conclusions | Summarise the main conclusions of the study | Abstract |
| 1e: Keywords | Include PPI, “patient and public involvement,” or alternative terms as keywords | N/A |
| **Section 2: Background to paper** | | |
| 2a: Definition | Report the definition of PPI used in the study and how it links to comparable studies | p.3 |
| 2b: Theoretical underpinnings | Report the theoretical rationale and any theoretical influences relating to PPI in the study | p.3 |
| 2c: Concepts and theory development | Report any conceptual or theoretical models, or influences, used in the study | p.3 |
| **Section 3: Aims of paper** | | |
| 3: Aim | Report the aim of the study | p.3 |
| **Section 4: Methods of paper** | | |
| 4a: Design | Provide a clear description of methods by which patients and the public were involved | p.3-4 |
| 4b: People involved | Provide a description of patients, carers, and the public involved with the PPI activity in the study | p.3 and Tables 1 and 2 |
| 4c: Stages of involvement | Report on how PPI is used at different stages of the study | p.3-4 |
| 4d: Level or nature of involvement | Report the level or nature of PPI used at various stages of the study | p.3-4 |
| **Section 5: Capture or measurement of PPI impact** | | |
| 5a: Qualitative evidence of impact | If applicable, report the methods used to qualitatively explore the impact of PPI in the study | N/A |
| 5b: Quantitative evidence of impact | If applicable, report the methods used to quantitatively measure or assess the impact of PPI | N/A |
| 5c: Robustness of measure | If applicable, report the rigour of the method used to capture or measure the impact of PPI | N/A |
| **Section 6: Economic assessment** | | |
| 6: Economic assessment | If applicable, report the method used for an economic assessment of PPI | N/A |
| **Section 7: Study results** | | |
| 7a: Outcomes of PPI | Report the results of PPI in the study, including both positive and negative outcomes | p.8-9 |
| 7b: Impacts of PPI | Report the positive and negative impacts that PPI has had on the research, the individuals involved (including patients and researchers), and wider impacts | p.8-9 |
| 7c: Context of PPI | Report the influence of any contextual factors that enabled or hindered the process or impact of PPI | p.8-9 |
| 7d: Process of PPI | Report the influence of any process factors, that enabled or hindered the impact of PPI | N/A |
| 7ei: Theory development | Report any conceptual or theoretical development in PPI that have emerged | N/A |
| 7eii: Theory development | Report evaluation of theoretical models, if any | N/A |
| 7f: Measurement | If applicable, report all aspects of instrument development and testing (e.g., validity, reliability, feasibility, acceptability, responsiveness, interpretability, appropriateness, precision) | N/A |
| 7g: Economic assessment | Report any information on the costs or benefit of PPI | N/A |
| **Section 8: Discussion and conclusion** | | |
| 8a: Outcomes | Comment on how PPI influenced the study overall. Describe positive and negative effects | p.8-9 |
| 8b: Impacts | Comment on the different impacts of PPI identified in this study and how they contribute to new knowledge | p.8-9 |
| 8c: Definition | Comment on the definition of PPI used (reported in the Background section) and whether or not you would suggest any changes | N/A |
| 8d: Theoretical underpinnings | Comment on any way your study adds to the theoretical development of PPI | N/A |
| 8e: Context | Comment on how context factors influenced PPI in the study | p.8-9 |
| 8f: Process | Comment on how process factors influenced PPI in the study | p.8-9 |
| 8g: Measurement and capture of PPI impact | If applicable, comment on how well PPI impact was evaluated or measured in the study | N/A |
| 8h: Economic assessment | If applicable, discuss any aspects of the economic cost or benefit of PPI, particularly any suggestions for future economic modelling | N/A |
| 8i: Reflections/critical perspective | Comment critically on the study, reflecting on the things that went well and those that did not, so that others can learn from this study | p.8-9 |
| **Abbreviations:** PPI – patient and public involvement | | |

**Supplementary Table 3: Guidance for reporting intervention development studies in health research (GUIDED) Checklist [13]**

| **Domain** | **Reported on Page No.** |
| --- | --- |
| 1. Report the context for which the intervention was developed | p.3 |
| 1. Report the purpose of the intervention development process | p.3 and Supplementary Table 9 |
| 1. Report the target population for the intervention development process | p.3 |
| 1. Report how any published intervention development approach contributed to the development process | p.3 |
| 1. Report how evidence from different sources informed the intervention development process | p.3-9 |
| 1. Report how/if published theory informed the intervention development process | p.3-4 |
| 1. Report any use of components from an existing intervention in the current intervention development process | N/A |
| 1. Report any guiding principles, people or factors that were prioritised when making decisions during the intervention development process | p.3-4 and Supplementary File 1 p.3 |
| 1. Report how stakeholders contributed to the intervention development process | pp.3-4 |
| 1. Report how the intervention changed in content and format from the start of the intervention development process | pp.3-9 |
| 1. Report any changes to interventions required or likely to be required for subgroups | Supplementary Table 9 |
| 1. Report important uncertainties at the end of the intervention development process | p.9 |
| 1. Follow TIDieR guidance when describing the developed intervention | Supplementary Table 9 |
| 1. Report the intervention development process in an open access format | N/A |

**Supplementary File 2: Post-Workshop Survey Findings**

| **Co-Design Experience Evaluation, mean (SD)** | | |
| --- | --- | --- |
|  | **Round 1, n=23^B^** | **Round 2, n=17**^C^ |
| Organisation and facilitation of session | 4.81 (0.40) | 4.65 (0.61) |
| Understanding of session objectives | 4.23 (0.81) | 4.59 (0.62) |
| Opportunity to shape and contribute to discussion | 4.76 (0.44) | 4.53 (0.63) |
| **Workshop Round 1 Quantitative Survey Findings (%)** | | |
|  | **Group 1 (n=8)** | **Group 2 (n=12)** |
| “I/my loved one/patients was/are given adequate information/education regarding the potential symptoms I/they may encounter throughout the lung cancer surgery journey”  Strongly agree  Agree  Neither agree nor disagree  Disagree  Strongly disagree | 38  25  25  13  0 | 0  17  33  50  0 |
| “I/my loved one/patients was/are given adequate information/education regarding how to manage these symptoms”  Strongly agree  Agree  Neither agree nor disagree  Disagree  Strongly disagree | 25  38  25  13  0 | 0  8  42  50  0 |
| “I/my loved one/patients was/are given adequate information/education regarding the importance of physical activity and exercise throughout the cancer journey”  Strongly agree  Agree  Neither agree nor disagree  Disagree  Strongly disagree | 38  38  0  25  0 | 8  8  17  67  0 |
| How confident did you/your loved one feel to exercise/be physically active after undergoing lung cancer surgery? (Group 1)  How confident do you feel providing education around exercise/physical activity to patients undergoing lung cancer surgery? (Group 2)  Very Confident  Confident  Neutral  Unconfident  Very Unconfident | 13  63  25  0  0 | 25  50  17  8  0 |
| When is the optimal time to receive pre-operative education/advice?  At time of diagnosis  During pre-admission clinic  During a dedicated education session (e.g., extra appointment)  In the hospital just prior to surgery  Other^D^ | 50  25  0  0  25 |  |
| When is the optimal time to receive post-operative education/advice?  In hospital after surgery  At the time of hospital discharge  1 week after hospital discharge  2 weeks after hospital discharge  2-4 weeks after hospital discharge  4-8 weeks after hospital discharge  >8 weeks after hospital discharge | 50  25  13  0  13  0  0 |  |
| When is the optimal time to commence post-operative exercise?  Immediately on hospital discharge  1 week after hospital discharge  2 weeks after hospital discharge  2-4 weeks after hospital discharge  4-8 weeks after hospital discharge  >8 weeks after hospital discharge | 63  25  0  13  0  0 |  |

^A^ Rated on a 5-point Likert scale (5= excellent, 1=poor)

^B^ Response rate = 82% (23/28)

^C^ Response rate = 71% (17/24)

^D^ Other: at any time prior to surgery (n=2)

**Supplementary Table 4: Themes and Subthemes from Semi-Structured Trigger Video Interviews and Workshop Round 1**

| **Theme/Subtheme** | **Quotations** | **Sub-Groups ^A^** |
| --- | --- | --- |
| **DIAGNOSIS & PRE-ADMISSION** | | |
| Theme 1: Diverse emotional and psychological responses to diagnosis | | |
| 1a: Feelings of shock, depression, terror, numbness, and fear of the unknown after an unexpected diagnosis | “I got the news that I had Lung cancer and it had spread, and as I mentioned, it was inoperable, um, and I was mortified. I really could not believe that what I was hearing was the truth.” – Patient Trigger Video Interview #3  “He called me in and told me that there was lung cancer there. Well, I was sort of a bit numb but I was like oh God, I knew something wasn't right.” – Patient Workshop #2 | Female patients |
| 1b. Fear around prognosis and survival | “Well, you know, when you hear that stage four cancer, I mean, obviously they think of the worst, you know, possibilities.” – Patient Interview #2  “And definitely, it's that fear of the unknown. It's, I guess, maybe the thought of dying and, you know, all those sorts of processes that go on.” – Professional Workshop #2 | Patients |
| 1c. Acceptance, realism and ‘go with the flow’ | “I personally went ahead, I trusted them, and I thought well as the old saying ‘Que Sera Sera,’ what can you do.” – Patient Workshop #3  “We don't get ahead of ourselves, just one step at a time, so I've been lucky with that otherwise I think I would have fallen apart.” – Patient Workshop #2 | Male patients |
| 1d. Influence of diagnosis on family, loved ones and relationship dynamics, concern for increased stress and new dependence on family | “I didn’t want to talk about it because I didn’t want to worry [my partner] or didn't want to worry part of my family.” – Patient Workshop #3  “...it [lung cancer diagnosis] did affect my husband more than it affected me. And my best friend, probably.” – Patient Trigger Video Interview #1 | - Patients - Caregivers |
| 1e. For some patients, an ‘operable’ diagnosis provides a glimmer of hope, and view the chance to re-evaluate their life and make healthy lifestyle changes as a ‘silver-lining’ | “He told me, he says, I mean, you're lucky.... he said, what you've got is cancer that's treatable and manageable. I'm thinking, well, that sounds like a bonus to me.” – Patient Interview #3  “When patients are offered surgery, which is the best chance of cure, then that sort of gives them a little bit of a glimmer of hope.” - Professional Trigger Video Interview #2 | Male patients |
| 1f. Implicit trust in medical team, gratefulness of medical advances (e.g., surgical interventions) and gratitude towards the healthcare system | “…Then my worry, again, is, ah, say is gone because say of the confidence, the trust that I have given to the doctors and the staff and also the state-of-the-art equipment of the hospital.” – Patient Trigger Video Interview #2  “I'm really grateful to the team...I feel like crying…I'm just so grateful for everything that's happened… we are very fortunate in this country.” – Patient Trigger Video Interview #1 | Male patients |
| 1g. Influences of stigma, self-blame and guilt associated with a lung cancer diagnosis, particularly on social isolation and mood | “If they have been a smoker in the past, maybe they feel really guilty about their lifestyle choices and regretful.” – Professional Workshop #2  “There's that stigma as well attached to it. And then when they get those comments from family or friends, they may self-isolate, and the mental health can also go down as well.” – Professional Workshop #1 | Professionals |
| Theme 2: The time between diagnosis and surgery is fast-paced and overwhelming | | |
| 2a. An overwhelming ‘whirlwind’ of tests, appointments, handouts, and information contributes to anxiety and stress levels | “Yeah, it was like a tornado, it just came through and then it was gone.” – Patient Workshop #1  “When patients are first diagnosed… they do have a lot of information to take in, and there's a lot of assessments, there's a lot of things that need to be done.” – Professional Trigger Video #2 | - Female patients - Professionals |
| 2b. Positive association of the speed to surgery, to ‘get it over with’ and have less time to ruminate and worry | “I'm grateful they got me in very quickly and that it was all rush-rush because I would hate to be sitting there anticipating the what ifs, if you didn't get in and then it had got worse.” – Patient Workshop #1  “It was really quick and that was the best thing because you didn't have time to really start worrying.” – Patient Workshop #3 | - Male patients - Patients from regional areas |
| 2c. Influence of overwhelm, anxiety and health literacy on ability to participate in and process education | “As for information, I’d just like to let you know, that really they didn't give me much information about what was happening, or maybe I was a bit in shock and I didn't understand it.” – Patient Workshop #1  “Other people just find it very overwhelming if you just start saying well you need to stop smoking, you need to exercise, you need to do this, and they're already in a very overwhelmed situation.” – Professional Workshop #1 | - Female patients - Professionals |
| 2d. Implementing prehabilitation and pre-operative education in the time between diagnosis and surgery is a challenge due to often short turnarounds and the overwhelming nature of this time | “I think preoperatively there just may not be a lot of time for you to do much more than just like see them once and give them some instructions on a few things they could do, but there often isn't a lot of time.” – Professional Interview #1  “…That it [preoperative exercise] just seems, especially in the midst of a lung cancer diagnosis, that seems insurmountable for many people.” – Professional Workshop #2 | Professionals |
| Theme 3: Patients have unmet educational needs before surgery | | |
| 3a. Some positive experiences of education around diagnosis and treatment plan (e.g., surgery) | “That's what I liked about it and they explained it in my language, everyday language, it wasn't all these big technical terms, and I found that really good, really helpful. So I suppose I went into surgery a lot calmer.” – Patient Workshop #2 | - Female patients - Patients from metro areas |
| 3b. A desire for more specific, credible/trustworthy information around diagnosis, upcoming procedure, and the road ahead to be provided to both patients and family members | “…I mean I was told my surgery would just be a couple of hours, so I sort of was a bit blasé about it. I didn't realise, and my husband didn't either, the seriousness of it. I thought with robotic surgery it would be very low-key surgery.” – Patient Workshop #1  “Make them aware of what's going to happen. But then don't put too much into it because they’ll only freak ‘em out. But then at the same time, don't leave them with not enough information. Say to them, do you understand? Please tell me, please ask me questions if you don't understand what I'm talking about.” – Patient Interview #3 | Female patients |
| 3c. Patients have diverse educational needs in the pre-operative period, with some searching for as much information as possible, and others taking a ‘the less I know the better approach’ | “But I’m the type of person that needs to know what's going on. It's like childbirth, you know the way through the canal, you don't panic.” – Patient Workshop #2  “Personally, the less I know the better it is for me.” - Patient Workshop #3  “Some people are very hungry for information straight away and they want to know everything straight up. Some people want to know a little tiny bit and then give them some brochures or resources to go away and absorb that. Some people don't want to hear about a lot of, you know, what's going to happen. They're very fearful of the surgery and they don't, you know, their ostrich in the sand kind of mentality.” – Professional Workshop #2 | Patients |
| 3d. Patients may choose to self-source education and resources, which proves to be a difficult journey in a sea of misinformation online, further compounded by health literacy levels | “…I think that it can be a little hard for some people to be able to find the info they want without being bombarded with rubbish.” – Patient Workshop #1  “The other thing was too, I was Googling and if I didn't Google I would have known nothing… But really there's not a lot of information unless you actually do research yourself.” – Patient Workshop #1 | - Female patients - Patients from metro areas |

| 3e. Some patients receive information/have an awareness re: about pre- and rehabilitation, but require more education, context, and skill building/enablement regarding what constitutes exercise, how to exercise/rehabilitate, how to remain active and modify program when symptoms feel insurmountable; and the “why” regarding the evidence and benefits | “I think it would also be useful for someone like me, but maybe not everybody, but to have an idea of the best way to rehabilitate yourself afterwards is, because then you can focus your mind on that rather than just sort of wallowing in the pain and wondering how you are going to get yourself better.” – Patient Trigger Video Interview #3  “I guess at a patient level some of the tangible benefits of doing [exercise] would be important as part of that pre-process so that they know why they're doing it.” – Professional Workshop #1 | Nil |
| --- | --- | --- |
| Theme 4: Need for comprehensive pre-operative assessment | | |
| 4a. Clinician beliefs around the important elements of a comprehensive pre-operative assessment, incorporating physical, nutritional, comorbidity, health status, and mood assessments | “Symptoms, so breathlessness, fatigue, like mental health and emotional functioning as well, what else, physical assessment of what their exercise capacity is like, and any pain and any range of movement issues they might have, and nutrition screening, so sort of all the multidisciplinary stuff.” – Professional Workshop #3  “I think I guess the first thing would be to understand the individual patient's current level of function, what their actual fitness is in order to say well look, is this something that they just need to try and maintain their level of activity in the lead up to their procedure or treatment, is there something that actually we could improve.” – Professional Workshop #1 | Professionals |
| 4b. Pre-operative assessment facilitates a better understanding of patients’ needs going into surgery, and unlocks the potential to intervene early for modifiable barriers and co-morbidities that may influence recovery/post-op course | “It probably goes back to why it's probably really important to have a good assessment to identify which people will need some supervision and support and which ones won't.” – Professional Workshop #3  “If they're having a lobectomy, they're going to lose lung function, so if we can do anything to optimise that beforehand, um, global rehabilitation, inspiratory muscle training who knows.” – Professional Trigger Video Interview #1 | Professionals |
| 4c. An important opportunity to provide much needed preoperative education, build capability to exercise, and to set expectations for post-operative journey | “So just building their confidence and understanding what their beliefs are [around exercise] a little bit to sort of help them through that period.” -Professional Workshop #1  “I think if they had an opportunity to meet with somebody like a physiotherapist… I think even a one-off meeting where they have time to sit and talk with somebody about how they can best prepare themselves to get through the operation well would probably be pretty valuable.” – Professional Interview #1 | Professionals |
| **INPATIENT HOSPITAL ADMISSION** | | |
| Theme 5: Inpatient rehabilitation focuses on respiratory optimisation and facilitating timely hospital discharge | | |
| 5a. Pain, breathlessness, nausea/vomiting, poor sleep, and other complications can be barriers to early mobility, and many patients feel unprepared for the reality and severity of post-operative symptoms | “Well, I didn't really sleep after the surgery. Because I was in enormous amounts of pain” – Patient Trigger Video Interview #1  “Dreadful. Pain. Yeah. Painful and hanging all the tubes in and out and so, this kind of general discomfort.” – Patient Interview #2 | - Female patients - Professionals |
| 5b. Experiences of reduced autonomy and independence, a new sense of vulnerability, and a desire for normalcy, early after surgery | “Even in the first few days you've got to buzz just to go to the toilet… I thought well bugger it, I'm not going to push a buzzer if I need to go to the toilet now, I'll just unhook it [my chest drain] and off I go… you’re just dependent on them at all times.” – Patient Workshop #1 | - Female patients - Patients from metro areas |
| 5d. The focus of ward-based rehabilitation interventions tends to be mobility, reducing the risk of PPCs, and facilitating an early discharge | “We're so focused on getting them through this acute phase and surgery that we maybe lose track of the bigger picture.” – Professional Workshop #3 | Professionals |
| **HOSPITAL DISCHARGE** | | |
| Theme 6: Need for additional education to support hospital discharge and recovery | | |
| 6a. Family/caregivers are not appropriately trained or supported to provide carer role on discharge, leading to anxiety and fears re: burdening loved ones | “There was a lot of anxiety, a lot of worry, for my partner, and then a lot of responsibility because when I came home from hospital, she was the one who had to do everything… Um, so yeah it puts a huge responsibility on people who are, just not trained for that… to suddenly be looking after somebody who is in a vulnerable situation and has had a serious surgery.” – Patient Trigger Video Interview #3 | - Female patients - Patients from metro areas |
| 6b. Barriers such as pain, nausea, and medication side-effects such as constipation/nausea/vomiting limit the effectiveness and appropriateness of providing discharge education on the ward | “…they've had surgery on the wards or whatnot and we know that they're in pain and they're on different medications that are affecting their cognition and their concentration and those things as well. That's the time that we tend to deliver education... But it's probably not the right time for them because it's - I know if I'm unwell like the last thing I want to hear if I've had surgeries is someone giving me education.” – Professional Workshop #3 | Professionals |
| 6c. Some patients are given verbal and/or written education re: exercise and rehabilitation at hospital discharge, but it often lacks context on the ‘how’ and ‘why’ to exercise | “I didn’t have any...I didn’t have any guidance on what to do, to rehabilitate myself so initially I rested.” – Patient Trigger Video Interview #3  “I think - I don't know, just from our service perspective I don't think the patients get much information at all about physical activity and exercise. I think they get told to just do stuff, maybe, like just stay active, but most patients don't know what that means or what they should be doing.” – Professional Workshop #3 | Nil |
| Theme 7: Perceived drop-off in care and support post-discharge can lead to feelings of abandonment | | |
| 7a. A lack of continuity, limited awareness of available services, and difficulty accessing support after hospital discharge leaves patients feeling deserted and isolated, and can force them to seek external care | “It was hard to get a hold of doctors at times, this was the second time around, the first time was okay. I ended up… I had to ring the doctor on call just to come out here and do it.” – Patient Workshop #1  “From there, though, where we fall down, I think, is that we don't really have a pathway that we can then send them, you know, onwards, we say, sort of, you know, see you later and that's about it.” – Professional Workshop #2 | Female patients |
| 7b. The current model of follow up is inadequate compared to other health conditions, does not effectively or objectively monitor progress and adopts a ‘reactive’ approach to management | “Like when we had our babies, soon as you went home you had a midwife used to come to your door to see how you were doing. On the phone, anytime you could ring, they would ring you, they’d come and see you every second day to see how you're going...” – Patient Workshop #2  “I feel like they maybe come back as an outpatient and then when they report that maybe they're not doing so well, then they say oh well actually maybe you should do X, Y and Z or maybe you need to see so and so about, you know, your mental health or - and so potentially those things are only brought up at a later time when they're actually saying I'm having a problem instead of knowing those options before the problem develops possibly.” – Professional Workshop #3 | Professionals |
| 7c. A strong desire for a more formalised, post-discharge ‘touch point/lifeline’ for monitoring, follow up and support, where continuity and connection is emphasised (i.e., repeated contact with a familiar HCP) | “So, to have someone that knows professionally that knows what you’re going through, is good to have that sort of support I think, just ‘til you get your mental and physical strength back.” – Patient Workshop #2  “So, I joined your research team and so I did get the call once a week which was good from [the physio]. But that really was the only lifeline I had.” – Patient Workshop #1  “The other thing that they [patients] talked about was still being under the umbrella of healthcare from their oncology or their surgery team and just how important it was for them to maintain that connection.” – Professional Workshop #3 | - Female patients - Patients from regional areas - Professionals |
| **LONG-TERM RECOVERY** | | |
| Theme 8: Experiences of persistent symptoms and difficulty returning to ‘normal’ | | |
| 8a. Impacts of neoadjuvant and adjuvant therapies particularly with side effects such as fatigue, N&V, immunosuppression | “The chemo I had a problem. The first dose I totally lost bladder control, talk about embarrassing. Then the last, the sixth dose of chemo made me really sick, and I got nausea tablets for that.” – Patient Workshop #2  “I, during chemo and radio I developed a fungal infection. After the surgery, that fungal infection got worse and in fact it...it grew in my trachea and my mediastinum and after...and this wasn't too long after surgery, that all of this really became quite serious” – Patient Trigger Video Interview #3 | Patients undergoing neoadjuvant and adjuvant therapy |
| 8b. Persistent breathlessness and other respiratory symptoms impact daily life | “Yeah, well, just running out of puff, you know. I can't do what I used to.” – Patient Interview #2  “As I say initially I got a lot of - I just felt a bit breathlessness and chest tightness, just not quite right, as if it’s suffocating a bit.” – Patient Workshop #3 | Nil |
| 8c. Experiences of unexpectedly severe fatigue, debilitation, reduced strength and exercise tolerance and influence on function/daily life | “I was tired, I was so tired, I would sit down and I never - for me to go to sleep at night’s even hard, I would just go to sleep and in the day.” – Patient Workshop #2 | - Female patients - Professionals |
| 8d. Experiences of surprisingly significant and/or persistent pain and its influence on daily function | “But the pain is incredible. As I said I've still got pain now, it's probably only just starting to ease off now, and I would have said that the pain’s pretty horrific.” – Patient Workshop #1  “Um, pain can be a thing that they don't necessarily quite expect at times, given they're in hospital where they have access to...you know, being asked and prompted about analgesia, and then going home and they have to do that themself.” – Professional Trigger Video Interview #2 | - Female patients - Professionals |
| 8e. The frustrating experience of new functional limitations and reduced ability to perform daily tasks, causing reduced confidence in abilities, a feeling of being ‘failed’ by one’s body, and a mismatch between expectations and actual physical capability | “I used to run for the bus with my daughter going to school, and there is no bloody way that I would do that now, I wouldn't even attempt to run for a bus, I mean bugger it, if it goes it goes, I'd rather sit there for 40 minutes than even try.” – Patient Workshop #1  “But it's never really been the same sort of ever since really, you know, because I get - I can't do the things I used to do… I used to be able to do a fair bit, but if I do something I feel physically sick, and I'm never like that, you know, so I've sort of got to stop and take time to get my breath back.” – Patient Workshop #2 | Patients |
| 8f. Experiences of common and rare post-treatment complications including post-operative infections, pleural effusions, pneumothoraxes, and rare musculoskeletal complications and their influence on healthcare utilisation, breathlessness, and functional decline | “But a few days later this tightness in the chest… because there is fluids and because I feel difficult to breathe, got a pain.” – Patient Workshop #3  “But I've also had - with the cancer, it sent toxins to my ankles, so I’ve got HPOA and I'm just living on Endone.” – Patient Workshop #2 | Female patients |
| 8g. A new dependence on family support and feeling a burden to family and the impact of change in role identity | “Then, you know, you've - well especially for you, you’ve got your family around that - you know, you're not able to do all those things that you used to be able to do, so for them it’s sort of difficult for them to sort of see you like that as well.” – Patient Workshop #2  “And that's also asking for help. Some people find it quite challenging. I guess it's taking on that patient role now where maybe they've been a mother, or a father and you know they're having to have that much more support.” – Professional Workshop #2 | - Female patients - Professionals |
| Theme 9: A lack of education leads to difficulty managing symptoms and contextualising recovery | | |
| 9a. Patients are given little-to-no guidance on what symptoms they may experience and how to manage them, and the presence and severity of symptoms can be shocking | “...I can't remember, really, being told what the implications of lung cancer treatment would be.” – Patient Trigger Video Interview #3  “I still sit here, and wonder will I - I don't think I'll ever be the same again. I certainly wasn't prepared to work that one out myself, which I had to do.” – Patient Workshop #1  “I feel like they haven't really been given any strategies, they've just thought well I've got this diagnosis and that explains it so that's that, when there are actually things that could be done to help those symptoms, but I don't know that they get a lot of that information.” – Professional Workshop #3  “These patients are often told you won't have long standing breathlessness, your lung function will only reduce by 5% or whatever, their pre-op, you know, they do have persistent breathlessness nine months post. So, it can be a little bit confronting.” – Professional Workshop #2 | Nil |
| 9b. Without education on potential and likely symptoms, patients struggle to contextualise their experiences against what is ‘normal’ or ‘abnormal’ and desire validation regarding symptom normalcy | “Being a spot to ask questions, you know, is this normal, am I recovering the right way, are my symptoms normal, am I managing them correctly, to normalise their recovery process.” – Professional Workshop #3  “But it’d be nice to know if – before, they said well that’s normal…You might have this, you might have this side effect and that’s normal, that’s fine.” – Patient Workshop #2 | - Female patients - Professionals |
| 9c. A common experience of mismatched expectations versus reality regarding realistic recovery and the importance of education and setting realistic timeframes/ expectations for the recovery journey | “I had to say, I think I had an unreasonable expectation that...that, once I'd recovered from the surgery, my capacity to breathe would be much the same as it had been before the surgery.” – Patient Trigger Video Interview #3  “I've always been pretty physically well, and with this one I just thought it would go away, I know that sounds really naïve, but I thought I would recover pretty much the same, I would be the same as I was before and just minus a bit of my lung. It's certainly not like that at all.” – Patient Workshop #1 | - Female patients - Professionals |
| Theme 10: Recovery should focus on patients’ goals and ‘getting back to normal’ | | |
| 10a. Focus on promoting functional independence, return to as normal life and work | “Just normal function. You're looking after your kids, doing those kind of things and resuming the role in your family that you had prior to surgery. So being the person that you were before and not being defined by disease or a condition.” – Professional Workshop #2  “For me rehabilitation is healing. That is one way or another about preparing the patient back to normal life.” – Patient Workshop #3 | Nil |
| 10b. Focus on transition to self-management, facilitate patients to take ownership of their recovery | “… you know, I had this surgery, I had lung cancer, so you are just waiting on it again to get worse and then I'm given a sentence, even with surgery, you know, is a possibility it’s a bad one I think. So, rehab was really helping you to really get back to yourself.” – Patient Workshop #3  “…We're providing I guess that rehab recovery phase of supported care to get them to achieve their goals and manage their symptoms, but then the ultimate goal is really to teach them how to self-manage as well.” – Professional Workshop #3 | Nil |
| 10c. Importance of collaborative goal setting, using meaningful outcomes and adopting a survivorship model | “I suppose the biggest thing too is you find out off the person where they want to end up… So, getting people to find their happy place.” – Patient Workshop #2  “… Not just like improving on a six-minute walk test but, you know, can they walk out to the mailbox, can they walk from a parked car to their favourite café.” – Professional Workshop #1  “Maybe we need to focus more on an experience and confidence type survivorship model.” – Professional Workshop #2 | Nil |
| Theme 11: Diverse levels of recovery and acceptance/adjustment | | |
| 11a. Experiences of coming to terms with and recalibrating to new functional limitations, and a belief that some reductions are ‘inevitable’ due to the nature of the surgery and ageing | “I don’t think that it's because I had the operation on my lung that I can’t. I think it’s because I'm older, I’m old, that’s why I can walk as far as I could walk two years ago or five years ago or whatever.” – Patient Workshop #3  “Patients just sort of accept it and say well I've got lung cancer so of course I'm breathless and I'm fatigued.” – Professional Workshop #3  “Well, now I would prefer a little bit more energy, but I know what it is. I accept… my limitations doesn’t worry me.” – Patient Interview #2 | - Male patients - Professionals |
| 11b. Experiences of grief towards previous functional levels and daily activities that are no longer achievable, and the influence of impairments and symptoms on identity/sense of self and locus of control | “…you're grieving for half your body's gone and you're grieving for the lifestyle that you didn't have.” – Patient Workshop #1  “I think that's one of the worst things is not having that, you know, that activity energy that you used to have” – Patient Workshop #2  “I used to do a lot of work for customers all the time, you know, and someone else is doing that work there now and I just wish it was me.” – Patient Interview #1 | Patients |
| 11d. Some patients experience a straight-forward, uncomplicated post-operative course with few or no persistent symptoms | “But a week or two later I was in the operating, all fine, next day or the day after I was home, pretty much doing – or even better than what I was beforehand.” – Patient Workshop #3 | Male patients |
| **ACROSS ALL TIMEPOINTS** | | |
| Theme 12: Importance of flexibility and individualisation of programs | | |
| 12a. There is no ‘one-size fits all’ approach to rehabilitation and education for this population, and models should strive to provide flexible options that adapt to patients’ needs, goals, geographical location, age, co-morbidities, and pre-existing exercise behaviours. | “I don’t know, it just probably all comes down to learning, helping everyone diagnose the ailments they’ve got. I don't know. Because everyone's different, not everyone's gonna have the same ... I wouldn’t think anyway.” – Patient Interview #1  “…Do they need to sort of be referred to something like a pulmonary rehab, you know, have they got goals, and would that be the right setting for them, versus just giving them education on a graded walking program and, you know, go at your own pace and monitor yourself. I think it could be pretty flexible.” – Professional Workshop #3 | Nil |
| 12b. Patients could be stratified to receive different ‘levels’ of support, supervision and education based on their wishes, needs, level of function, goals, and health status (e.g., comorbidities, neo/adjuvant treatment, frailty, etc.) | “Well if the patient is quite fine and could easily understand what was given in that pamphlet or whatever, that will be good for him as long as he understand it. But the problem is there are some that could not understand or apply what was written in that particular pamphlet or booklet. Then it has to be assisted personally and be advised to go in a program maybe.” – Patient Workshop #3 | Nil |
| 12c. Accessibility and equity should be key considerations when designing future programs e.g., considerations of health literacy, technological access and literacy, and language | “Well, I mean a phone call, that’s about all that I can receive. But if they start with Zooming and stuff like that forget about it. I prefer face to face because then I can express myself, and if I can’t hear what they’re saying I can say speak a little bit louder.” – Patient Workshop #3  “A lot of our patients are still a little bit older, and I don't think would like do really well with an eLearning module. You know, like there's a – like not a nonzero number of them that don't have a mobile phone, you know, like definitely do not use the internet.” – Professional Interview #1  “Well it's a bit hard understanding medical terms too, you know, so it needs to be sort of explained to us in a bit of a lay terms way so we understand.” – Patient Workshop #2 | Nil |
| 12d. Travel, transportation and geography are barriers to participating in programs, particularly for patients living in rural and regional areas | “There's no reason why we should be subjected to that postcode lottery of, you know, we're living here, we can't necessarily get up and move and relocate to somewhere else so, you know, let's be a little bit more flexible in the way that we deliver things.” – Professional Workshop #3  “I think it's better if it's near where you live. Because my problem is to drive all the way in here.” – Patient Workshop #3 | - Patients from regional areas - Professionals |
| 12e. Influence of employment/work schedule and financial situation on participation in rehabilitation | “I don’t sort of look after myself in that respect because I'm busy working. You know?” – Patient Interview #3 | Professionals |
| Theme 13: Influence of pre-existing habits and beliefs surrounding exercise | | |
| 13a. Viewing rehabilitation/exercise as important/beneficial, and having goals to return to a previously active lifestyle and/or self-efficacy/skills relating to exercise, are important enablers to participating, and vice versa | “I think, you know, just say some people come into a lung cancer diagnosis with a background of being quite inactive for a large part of their lives and other people who may have been quite physically active, and so they're quite different people with quite different needs.” – Professional Workshop #1 | Nil |
| 13b. Some patients’ beliefs regarding exercise and physical activity are barriers to participation before and after surgery– e.g., “I am already active at work or around the house” or “I am too fit/active/unrestricted to require rehabilitation/exercise” | “No, no, it’s not that I don’t want to do it [exercise]. I feel that I don't need to.” – Patient Workshop #3  “I'm on my feet 12 hours a day so I'd regard that as enough exercise. You know, and there's only small distances that I may have to walk but I'm upright 12 hours a day. So, I don't, I very rarely sit down.” – Patient Interview #3 | Male patients |
| Theme 14: Lack of opportunities for peer support | | |
| 14a. For some patients, there is an unmet desire for peer-support which can help patients contextualise their own recovery, make sense of what is normal, build self-efficacy, and hear from the ones who ‘get it’, which reduces feelings of isolation and aloneness throughout journey | “For me I was reading so many different accounts like on Reddit and it's just a general form of people's experience. I think most people – I know most people are different, they're going to recover and deal with this differently, so I was trying to get a big - like a consensus of it basically and that I did have to find myself.” – Patient Workshop #1  “You think you're not doing too well, but when you're talking to someone that's been through a similar thing you realise you're not so bad after all. That can be quite a positive thing.” – Patient Workshop #2 | - Female patients - Professionals |
| 14b. Patients are interested in opportunities to hear from previous patients, including via videos, especially prior to surgery to learn more about self-management, how to prepare, and the journey that is to come | “I think the video about people who have gone through the surgery, how they progressed from there may help some people in their recovery. To just see how the other people do, having the same problem, or even worse, and they are recovering and that may give them a boost in their recovery, to their future outlook.” – Patient Workshop #3 | - Female patients - Professionals |
| Theme 15: The inextricable links between mental health, mindset and recovery | | |
| 15a. Influence of mental health on recovery and participation, and the influence of self-efficacy and mental mindset on recovery and readiness to participate in exercise/rehabilitation e.g., optimism, determination, desire to regain control over condition | “Then, you know, there's depression, there's all sorts of things, you know, that come into it, you know.” – Patient Workshop #2  “Because if you will continue worrying it will hinder your healing. But if you have a peace of mind and then you have inculcated it in the mind of the patient then for sure the healing will be faster.” – Patient Workshop #3 | Patients |
| 15b. Influence of diagnosis, impairments, and recovery on mental health, and the reduction in confidence and self-efficacy related to symptoms and impairments | “I think that psychological support at this sort of phase and in the more sort of medium term is very important about how people adjust to their new sort of sense of self, their identity, how it changes...” – Professional Workshop #1  “When something has happened to your body that is really quite serious, um, I lost confidence in how far I could push myself in order to get fit again” – Patient Trigger Video Interview #3  “I understand that some people even after surgery is experiencing trauma, maybe they think of ah, what happen to them so that will lead to worry, again. Number one enemy here is worrying. So all you have to do is build up confidence.” – Patient Trigger Video Interview #2 | - Female patients - Professionals |
| 15d. Patients feel unprepared for the psychological impact of lung cancer treatment, and there is a distinct lack of mental health resources and supports offered and available for patients across the continuum | “I just was not - I didn't even know how to explain it. I wasn't prepared for any of the mental state that you might be in at all, yeah, no, not informed.” - Patient Workshop #1  “Not so much sort of - like all these ads on TV, you know, they say about, you know, counsellor to call but, you know, I thought about that but I thought well what – who - I never really want to do it, I don’t want to talk to someone…it'd be nice to have someone that you could actually talk to in person that you sort of had a, you know, like a connection with… someone that you have regular sort of chats with.” – Patient Workshop #2  “Lots of our patients very openly say to me how much they've really struggled with the mental health side of things even when they've had like a relatively early lung cancer with a good prognosis and not needing chemotherapy and everything, it's a lot for people.” – Professional Interview #1 | Nil |
| 15e. Some patients view their journey as ‘traumatic’, particularly the hospital ward stay, which is associated with a frantic desire to ‘get out’ of hospital and a fear of readmission/returning to hospital | “I don't know how [Patient] did it having two surgeries, I think that would be really quite scary. I'm hoping I never have to experience it again in my life, it was really traumatic.” – Patient Workshop #1  “It was getting to the point where I was, needed to get home, for my own sanity” – Patient Trigger Video Interview #1 | Female patients |
| 15f. Time away from work and financial stressors further exacerbate feelings of anxiety/stress, and impact patients’ sense of self and identity | “I went back to work fairly quickly more for mental health issues and money and that sort of thing because no one's paying you to stay home, once your sick leave’s gone.” – Patient Workshop #1 | Nil |
| Theme 16: The influence of family/loved ones and culture | | |
| 16a. The positive influence of social support, and conversely the negative influence of isolation, on motivation, mental health, and recovery | “Because my friend is also retired now, so two of us go for walking, and we walk up the hill a bit, down hill a bit.” – Patient Workshop #3  “And then when they get those comments from family or friends, they may self-isolate and the mental health can also go down as well.” – Professional Workshop #2 | Nil |
| 16b. Importance and benefits of involving family members in education, and upskilling family members to take role of motivator/enabler for rehabilitation and recovery, while maintaining an awareness of different patients’ needs and desires for speaking or ‘burdening’ their loved ones regarding their diagnosis | “I think getting family on board can be such an asset” – Professional Workshop #1  “They told my husband a lot more than what they actually told me which was a little bit insulting in some ways, because I did get it.” – Patient Workshop #1  “Oh look, there's no doubt that a lot went straight over my head, and [my partner] was with me all the way, he was there all the time so I could ask him questions afterwards, yeah, been really good like that.” – Patient Workshop #2 | - Female patients - Professionals |
| 16c. Barriers to recovery related to family and cultural factors, particularly if not skilled/educated regarding the importance of physical activity e.g., encouraging rest | “Different cultures would have different expectations on what you do with a cancer diagnosis. And I think maybe overcoming those cultural barriers would be quite challenging, especially if maybe family don't want them to exercise or want them to, you know, wrap them in cotton wool and look after them and maybe don't realise how important it is to let them be active and encourage them with their activity.” – Professional Workshop #2  “You have to consider the culture and background of the person… so what's the culture, that will increase their ah, their confidence and trust.” – Patient Trigger Video Interview #2 | Nil |
| Theme 17: Preferences for optimal program design differ, but continuity and follow-up should be emphasised | | |
| 17a. There are diverse opinions regarding the optimal delivery and content of rehabilitation interventions, highlighting the importance of flexibility | “Yeah, I chose website…But I appreciate websites aren't necessarily accessible by everybody. You know, it's a bit of a trade-off isn't it.” – Professional Workshop #3  “I prefer the personal contact like so you there and you’ve put it on YouTube and say right, that’s, you know, that’s there for you, this is how you do it, and then you sort of - you're not going into it thinking oh am I doing this right or wrong.” – Patient Workshop #2  “Maybe there just needs to be a decent website where it's coming from you that you're giving the right information….” – Patient Workshop #1  “I think having Lung cancer specific programs…does provide that space and people, I find, are more likely to engage in rehabilitation if they're, you know, if they're in their community of patients, or other people, who have gone through a...a similar thing.” – Professional Trigger Video Interview #3 | Nil |
| 17b. New programs should consider pragmatic approaches to utilising pre-existing resources and services | “I think in the real world, you know, we're getting more and more lung cancer patients and not particularly more staff, so it's very much about trying to work together and integrate it into existing services so that we can use what we have at least to get started until potentially resources pick up.” – Professional Workshop #1 | Professionals |
| 17d. Importance of a multi-modal, survivorship-model approach, incorporating adjuncts such as smoking cessation, diet, mental health and medication management support | “I think in an ideal world that would be beautiful to be able to have some sort of one stop shop of help with nutrition and exercise and smoking cessation.” – Professional Workshop #1  “I think a probably a multimodal approach would be good for them….Um, if appropriate I think lifestyle education, emotional and mental health check ins. Diet, if required.” – Professional Trigger Video Interview #1 | Professionals |
| 17e. Formalised contact/touch points from a familiar HCP incorporating opportunities to ask questions are key regardless of the mode of intervention delivery, with an emphasis on the importance of clinician continuity to build trust | “I think there is a phone number you can ring but, yeah, you shouldn't have to be relying on, like [Patient] said, ringing a - you should be able to have a contact that's there without outsourcing.” – Patient Workshop #1  “I think it'd be a fabulous idea to have continuity of a person because they can build that trust and then it also kind of encourages them to continue on with it, I think if they know someone.” – Professional Workshop #1 | - Female patients - Professionals |
| 17f. A desire for follow-up/check in to monitor patients’ recovery and interaction with education at multiple timepoints across the continuum | “So, after the surgery and physio was checking on me, I go for – walking how many steps every day, increasing gradually. I think that really helps me in a way disciplined so that I need to go and, you know, to go for a walk now.” – Patient Workshop #3 | Nil |
| 17g. Patients tend to view home-based interventions as preferable to centre-based programs, incorporating exercise around their usual environment such as walking, and face-to-face contact with HCPs to monitor progress and correct exercise technique | “… So it's different people's different needs where may be really good for the physio to assess them at home if possible.” – Patient Workshop #3  “From my perspective still in the hospital, [laughs] only because - or an outpatient service or something, because I don't think we've quite yet figured out how to properly assess people at home. But I think that would be a nice option to be able to assess people at home” – Professional Workshop #3 | - Patients - Caregivers |
| Theme 18: Clinician training and upskilling | | |
| 18a. Potential avenues to upskill and empower other disciplines and junior staff to provide rehabilitation education and advice to improve access and availability | “I think an online model would be the way forward. I think it'd be great if it could be factored into part of an induction or something that just showed that again it's embedding it into the importance of living well and trying to optimise patients… something great that you could just give health professionals the confidence in giving an overall kind of education to a patient about exercise and breathing rehabilitation.” – Professional Workshop #1 | Professionals |
| 18b. Need for guidance and resources for clinicians regarding current evidence and optimal patient management | “I think for me it's definitely the confidence, so I don't have a whole lot of experience with lung cancer patients when they - from after diagnosis and then before they receive treatment, after, during, so I think that's a big barrier to being able to implement something.” – Professional Workshop #1  “But now, you know, I have caught up with a bit more and largely it was a course that I did…that really opened my eyes to prehab and everything that is involved and the research that's been out there..” Professional Workshop #2 | Professionals |
| 18c. Acknowledgement of the current gaps between evidence and practice, particularly regarding prehabilitation | “And I think that's one of the large barriers to this section is we know the evidence is there. Some of us maybe know the evidence is there [laughing], but it's not in the general knowledge of physios. It's not in the general GP's repertoire. They're not offering it to their patients maybe because they know about it, and they don't know who can provide that.” – Professional Workshop #2 | Professionals |
| Theme 19: Limited resources and established models of care are current barriers | | |
| 19a. Limited resources, lack of dedicated programs, and long waitlists to existing services limit the provision of gold-standard pre- and rehabilitation in this population and limit possibilities for integrated care post-hospital discharge | “I know the pulmonary rehab program that they're running, you know, has extended waitlists, and you know, trying to add lung cancer surgery patients into there, you know, it doesn't really fit with the model.” – Professional Workshop #2  “Particularly a lot of our patients come from rural areas, and they often have long waitlists or just lack of availability.” – Professional Workshop #3  “For me it's the lack of dedicated services.” – Professional Workshop #1 | Professionals |
| 19b. There is a lack of consistency in available care across the continuum of care and between health services, particularly in the public vs. private health systems | “But I think the barriers between public and private that I'm seeing quite a bit is the funding models are so different… there's not a lot in it for a private hospital to run these programs.” – Professional Workshop #2  “Certainly, down in [State] we just have a lack of resources for anything. So, there's no prehab.” – Professional Workshop #1  “I think there's a lot of disjointed care… they might have been diagnosed somewhere else and then they get referred into a surgeon somewhere and they get information from lots of different people.” – Professional Workshop #3 | Professionals |
| 19c. Referring patients to pre- and rehabilitation is currently a complex process, with barriers such as unclear and unstructured referral processes, relying on individual clinician decision making, poor awareness of available services, and lack of ‘ownership’ of the referral process | “It just seems like a lucky dip sometimes in terms of who gets referred and who doesn't.” – Professional Workshop #3  “…just that lack of understanding about who was responsible for making sure that that prehab conversation was happening… There just wasn't that sort of decision-making process in place.” – Professional Workshop #3 | Professionals |
| ^A^ Subgroups of participants that have contributed a significant proportion of quotes/codes to a subtheme. If ‘nil’, no primary contributing subgroup was identified for a sub-theme (i.e., all subgroups contributed significantly to sub-theme). | | |

**Supplementary File 3: Qualitative Findings Among Participant Subgroups**

Two themes (18 and 19) were comprised entirely of insights from Group Two. No themes were identified based solely on findings from Group One. Female patients were more likely to report unmet needs regarding education and symptom management. Female patients were also more likely to report higher symptom burden and a desire for supervised exercise. Male patients were more likely to report that their symptoms/ impairments were inevitable due to their age and/or diagnosis, or that they did not require rehabilitation support due to their activity levels or lack of symptoms. Patients who underwent neoadjuvant and adjuvant therapy were more likely to report more severe symptoms and side effects. Patients/caregivers living in both metropolitan and regional areas preferred home-based interventions where possible, however, those from regional areas were more likely to report barriers associated with digital literacy, digital access, and travel/transport.

**Supplementary Table 5: Current barriers to exercise/rehabilitation identified during data collection mapped to COM-B and TDF models**

| Capability | | |
| --- | --- | --- |
| COM-B Component | TDF domain | Current barriers |
| Psychological | Knowledge | - Lack of knowledge re: potential symptoms and their management |
|  |  | - Lack of knowledge re: what is ‘normal’ and ‘not normal’ throughout the disease trajectory |
|  |  | - Lack of knowledge re: importance/rationale of exercise and rehabilitation (e.g., fitness for surgery, recovering from surgery, reducing risk of PPCs) |
|  |  | - Lack of both patient and clinician awareness of available supports/avenues for follow-up care |
|  |  | - Lack of knowledge re: diagnosis and implications, surgical management and what is to come |
|  |  | - Reduced clinician education and awareness of evidence and best practice |
|  | Skills (Cognitive and Interpersonal) | - Poor digital literacy and inability to participate in Telehealth interventions for some individuals |
|  |  | - Lower health literacy reduces ability to understand and process education for some individuals |
|  |  | - Lack of family member capability and training to provide ‘caregiver’ role |
|  |  | - Reduced clinician knowledge of and ability to complete referral processes to access follow-up care |
|  | Memory, Attention and Decision processes | - Overwhelming ‘whirlwind’ of diagnosis and pre-operative timepoint reducing ability to accept education due to competing priorities and cognitive fatigue |
|  |  | - Lack of educational resources to refer to in their own time/at their own pace |
|  |  | - No desire for education – ‘the less I know the better’ |
|  | Behaviour Regulation (Habits) | - Pre-existing sedentary behaviours |
|  |  | - Limited ability to monitor own symptoms and recovery |
|  |  | - Lack of support to understand or break unhelpful habits |
| Physical | Skills | - Reduced exercise tolerance and fitness |
|  |  | - Persistent symptoms of pain, fatigue, and breathlessness |
|  |  | - Side-effects of neo-adjuvant and adjuvant cancer treatment e.g., nausea, fatigue, immunosuppression |
| Opportunity | | |
| COM-B Component | TDF domain | Current barriers |
| Physical | Environmental context and resources | - Isolation and reduced access to support/follow-up due to geographical location/rurality |
|  |  | - Limited flexibility and individualisation of available services |
|  |  | - Financial costs associated with attending rehabilitation |
|  |  | - Lack of available, appropriate rehabilitation services |
|  |  | - Long waitlists of existing services (e.g., Pulmonary Rehabilitation) |
|  |  | - Lack of available healthcare resources (e.g., funding, staffing and equipment) |
|  |  | - Constraints and goals of existing models of care/clinical pathways (e.g., focus on inpatient respiratory management and timely/safe discharge home) |
|  |  | - Inconsistent availability of programs and resources across different health services and in public vs. private settings |
| Social | Social influences | - Lack of continuity and follow up to allow development of therapeutic alliance between patient and HCPs |
|  |  | - Competing priorities e.g., caregiver and work responsibilities reducing perceived time available to participate in exercise/rehabilitation |
|  |  | - Stigma of lung cancer diagnosis |
|  |  | - Potentially unhelpful cultural and/or family approaches to recovery e.g., encouragement to prioritise rest |
|  |  | - Poor understanding of lung cancer symptoms and treatment side-effects from support network |
|  |  | - Lack of opportunities for contact with others with lung cancer |
| Motivation | | |
| COM-B Component | TDF domain | Current barriers |
| Reflective | Beliefs about capabilities (confidence) | - Lack of self-efficacy and determination/grit |
|  |  | - Inability to contextualise own recovery against what is ‘normal’ or ‘abnormal’ |
|  |  | - Grief and difficulty adjusting to new symptoms and functional impairments |
|  |  | - Belief that “I do not require rehabilitation” |
|  |  | - Reduction in own perceived abilities due to older age and/or presence of comorbidities |
|  | Social/Professional Role/Identity | - View of self as an ‘inactive/lazy’ person |
|  |  | - Lack of clinical oversight or ownership, unclear who is responsible for referral and follow up |
|  | Beliefs about consequences | - Declining referrals to support/follow-up due to lack of understanding of potential symptoms and impairments |
|  |  | - Belief that there is no additional benefit to structured exercise over regular daily activities e.g., gardening, walking or lifting at work |
|  | Optimism/pessimism | - Negative beliefs about prognosis and likely outcomes |
|  |  | - ‘Horse has bolted’ mentality re: prehabilitation – i.e., it is too late to start exercising between diagnosis and surgery |
|  |  | - Belief that the overall outcome cannot be changed – what will be will be |
|  | Intentions (I plan to) | - Limited follow up and oversight of rehabilitation/recovery |
|  |  | - Reduced motivation to exercise |
|  | Goals (I want to) | - Lack of support and monitoring to set and achieve goals |
|  |  | - Lack of prioritisation of fitness/exercise/recovery as a key goal in relation to other competing priorities |
| Automatic | Emotions | - Feelings of desertion and isolation from the health system after hospital discharge |
|  |  | - Worry, anxiety, and fear for the future |
|  |  | - Lack of enjoyment of exercise |
|  | Reinforcement | - Reliance on encouragement from family/friends to participate in exercise |
|  |  | - A vicious cycle of symptom aggravation (e.g., pain, shortness of breath) during exercise reinforcing sedentary behaviours |
| *Key:* Whole cohort voiced; Group 1 (patient/caregiver) voiced; Group 2 (professionals) voiced  Abbreviations: PPCs – post-operative pulmonary complications | | |

**Supplementary Table 6: Current facilitators to exercise/rehabilitation identified during data collection mapped to COM-B and TDF models**

| Capability | | |
| --- | --- | --- |
| COM-B Component | TDF domain | Current facilitators |
| Psychological | Knowledge | - Clinician-delivered education sessions and pamphlets – typically provided during surgical workup regarding diagnosis and upcoming procedure |
|  |  | - Access to pre-existing education materials online – e.g., advocacy organisations, hospital websites, and message forums |
|  |  | - Clinician knowledge and confidence to complete an assessment, provide education and prescribe exercise |
|  | Skills (Cognitive and Interpersonal) | - Adequate digital literacy to participate in Telehealth interventions and to self-source digital education for some individuals |
|  |  | - Adequate health literacy to understand and process education (both hospital-delivered and self-sourced) for some individuals |
|  | Memory, Attention and Decision processes | - View of self as somebody who needs to be informed to cope |
|  | Behaviour Regulation (Habits) | - Pre-existing active behaviours |
| Physical | Skills | - Pre-morbidly high exercise tolerance, fitness and muscle strength |
|  |  | - Absence of or few ongoing symptoms post-operatively |
| Opportunity | | |
| COM-B Component | TDF domain | Current facilitators |
| Physical | Environmental context and resources | - Implicit trust and gratitude for medical team and treatment |
|  |  | - Utilisation of opportunities for reimbursed/publicly funded exercise sessions |
|  |  | - Utilisation of existing services (e.g., Pulmonary Rehabilitation, Oncology Rehabilitation, and other public and private community services) |
|  |  | - Increased access to support/follow-up due to geographical location in metropolitan areas – ‘winning the postcode lottery’ |
| Social | Social influences | - Existing opportunities to access peer-support e.g., local cancer support groups and via advocacy organisations |
|  |  | - Family/loved ones providing motivation to exercise, discouraging rest and/or offering company during exercise activities such as walking |
|  |  | - Family/loved ones providing support and offloading patients’ responsibilities within home to facilitate recovery |
| Motivation | | |
| COM-B Component | TDF domain | Current facilitators |
| Reflective | Beliefs about capabilities (confidence) | - High levels of self-efficacy and determination/grit |
|  |  | - Belief that “I can participate in exercise despite barriers (e.g., age, symptoms, co-morbidities)” |
|  | Social/Professional Role/Identity | - View of self as an ‘active’ person |
|  | Beliefs about consequences | - Belief that participating in exercise/rehabilitation is an important part of recovery |
|  |  | - Positive perceptions of the importance of exercise for general health – “if you don’t use it, you’ll lose it” |
|  | Optimism/pessimism | - An ‘operable’ diagnosis improves mindset and positivity towards potential outcomes |
|  | Intentions (I plan to) | - Decision to incorporate exercise/physical activity into daily routine |
|  | Goals (I want to) | - Having goals to return to previous lifestyle – e.g., previous exercise routine, social or cultural outings, active hobbies, employment |
|  |  | - View of exercise and improving physical capabilities as an important tool to ‘fight’ lung cancer |
| Automatic | Emotions | - Keeping a positive mindset despite diagnosis and challenges |
|  | Reinforcement | - Benefit of encouragement from family/friends |
|  |  | - Feeling better, or having less symptoms, after completing prescribed exercises or going for a walk |
| *Key:* Whole cohort voiced; Group 1 (patient/caregiver) voiced; Group 2 (professionals) voiced. | | |

**Supplementary Table 7: Proposed intervention components mapped to the Behaviour Change Wheel intervention functions**

| Intervention functions | Proposed intervention components* | Incorporated into prototype |
| --- | --- | --- |
| Education | - Assessment of patients’ educational needs and readiness to receive education` | Y |
|  | - Comprehensive, individualised education across the trajectory incorporating in-person, online and paper-based models | Y |
|  | - Regular reinforcement of previously provided education | Y |
|  | - Development of an online education module for patients and clinicians | Y |
| Persuasion | - Provide education on the differences between activity in workplace/at home and structured exercise | Y |
|  | - Utilise patient-preferred language to refer to intervention – e.g., ‘fitness’ program rather than ‘exercise’ program to reduce influence of negative connotations and past experiences | Y |
|  | - Buy-in from medical/surgical teams to facilitate their encouragement of participation in rehabilitation | N |
| Incentivisation | - Education regarding the importance of and evidence for exercise to enable patients to build mental connection between exercise and positive outcomes/symptoms | Y |
|  | - Consider incorporation of activity monitoring tech where appropriate | Y |
| Training | - Exercise-based rehabilitation intervention with a focus on building physical capability (e.g., muscle strength, exercise tolerance) and exercise skills (e.g., self-management skills) | Y |
|  | - Training and guidance for caregivers and patients regarding supporting loved ones, and medication and wound management | Y |
|  | - Mental health support specifically focused on mindset, confidence and self-efficacy building | Y |
|  | - Empower and train caregivers/loved ones to support and monitor patients through their rehabilitation | Y |
|  | - Upskilling of clinicians (from both exercise/rehabilitation backgrounds and other disciplines) and enhanced dissemination of research evidence | Y |
| Environmental restructuring | - Implementation of a flexible model of rehabilitation across the lung cancer trajectory | Y |
|  | - Implement a formal screening for patient’s rehabilitation, educational and support needs | Y |
|  | - Creation of a ‘hotline’ or centralised access point to ask questions and ensure appropriate recovery | Y |
|  | - A checklist-style ‘check-in’ to evaluate patients’ engagement with program elements including education | Y |
|  | - Enhanced access to lung cancer nurse specialists | N |
|  | - Pragmatic utilisation of current programs and resources | Y |
|  | - Alter current clinical pathways and protocols to incorporate pre- and post-operative rehabilitation | N |
|  | - Incorporate a clear system to deal with/escalate patients with severe symptoms and/or mental health concerns (e.g., ‘traffic-light’ system) | Y |
| Modelling | - Incorporate stories from previous patients who have undergone successful rehabilitation and sharing of their experiences of common symptoms and setbacks | Y |
|  | - Access to support groups or peer-support for patients who desire this (consider via pre-recorded video and/or face-to-face/telehealth models) | Y |
| Enablement | - Formal collaborative, achievable goal setting | Y |
|  | - Regular follow-up with familiar/consistent clinician for motivation and support | Y |
|  | - Focus of rehabilitation and recovery programs on returning to ‘normal’ – resumption of previous social and workplace role | Y |
|  | - Continuity and follow-up from hospital service | Y |
|  | - Provide a realistic understanding of the journey ahead | Y |
|  | - Contextualisation of what is ‘normal’ | Y |
|  | - Provide opportunities to ask questions | Y |
|  | - Normalise and centralise mental health assessment and management throughout entire program | Y |
|  | - Pre-operative assessment +/- intervention for modifiable risk factors | Y |
|  | - Integration of smoking cessation education and support across the continuum commencing at time of diagnosis | Y |
| *Key:* Whole cohort voiced; Group 1 (patient/caregiver) voiced; Group 2 (professionals) voiced.  * Derived from current barriers and facilitators (Table 4 and 5) and/or directly from workshop/interview findings (Table 3). | | |

**Supplementary Table 8: Joint display of qualitative and quantitative findings, meta-inferences and interpretation**

| **Qualitative Findings (Related themes and quotes)** | **Quantitative Findings** | **Meta-inferences and Interpretation** |
| --- | --- | --- |
| **Themes:**   1. Patients have unmet educational needs before surgery 2. Need for additional education to support hospital discharge and recovery 3. A lack of education leads to difficulty managing symptoms and contextualising recovery   **Quotes:**   - “But he did inform me of how the surgery would be… really listened to me and informed me...” – Patient Workshop #1 - “Personally, the less I know the better it is for me.” - Patient Workshop #3 - “I can't remember, really, being told what the implications of lung cancer treatment would be.” – Patient Trigger Video Interview #3 - “I got a leaflet from the...that sort of explained it, but yeah not really… I got told not to Google... but I mean, of course you do, of course you research it, so yeah, I did most of it [sourcing education] myself.” – Patient Trigger Video Interview #1 |  | Group 2 (professionals) survey data had a high degree of convergence with the qualitative findings, whereas Group 1 (patients/caregivers) mostly reported receiving adequate education about potential symptoms. There was some degree of convergence with the findings of the Group 1 workshops, with some patients reporting they were provided with adequate education in the lead-up to surgery, particularly regarding their upcoming procedure. However, when expanding on these findings during workshops, patients reported significant unmet needs, and instances of self-sourcing education. An area of complementarity between the datasets emerged from sub-group analysis, which showed that female patients were more likely to report unmet education needs. Overall, male participants were much less likely to report unmet needs or high symptom burdens.  Potential sources of divergence include the low survey response rate (66% of Group 1), the ambiguity of the survey question, the findings of the qualitative sub-group analysis, and potential sources of bias (e.g., patients reported high levels of gratefulness for their medical care and may not have wanted to mark their care as ‘inadequate’, and some researchers did have prior research relationships with patients via their hospital institution).  By re-exploring these topics in Workshop Round 2, it became clear that participants from both groups felt that overall, current practices regarding pre-operative education are not adequate. |
| **Themes:**   1. A lack of education leads to difficulty managing symptoms and contextualising recovery   **Quotes:**   - “I still sit here, and wonder.. I don't think I'll ever be the same again. I certainly wasn't prepared to work that one out myself, which I had to do.” – Patient Workshop #1 - “I feel like they haven't really been given any strategies… when there are actually things that could be done to help those symptoms, but I don't know that they get a lot of that information.” – Professional Workshop #3 |  | Similar patterns of convergence, divergence, expansion and complementarity were seen for this domain. During the first round of workshops, patients (particularly female patients) reported feeling ‘left to their own devices’ to manage unexpected symptoms. We propose the same potential sources of divergence as listed above.  As above, given this divergence, these topics were re-explored in Workshop Round 2, during which both participant groups reported that current education around symptom management should be improved, and provided strategies for this that were incorporated into the intervention prototype. |
| **Themes:**   1. Patients have unmet educational needs before surgery 2. Need for additional education to support hospital discharge and recovery   **Quotes:**   - “I didn’t have any...I didn’t have any guidance on what to do, to rehabilitate myself so initially I rested.” – Patient Trigger Video Interview #3 - ‘They didn't say anything to me about how I should go about anything after that. Just generally care of the wound itself.” – Patient Workshop #1 |  | Again, we identified good convergence with Group 2’s quantitative and qualitative findings, juxtaposed with a level of divergence within Group 1.  The workshops and interviews expanded on the survey data and revealed that while some patients were told to some exercise during their journey, they were not provided with enough explicit education around the ‘why’, ‘how’, or ‘when’ to enable them to effectively participate in exercise.  When considering the complementarity of the data sets, the subgroup patients who were not involved in post-operative exercise programs were more likely to report these unmet needs. Additionally, female patients were more likely to report inadequate education.  Along with the potential sources of divergence already proposed above, a proportion of the patients involved in this study did participate in a post-operative physical activity research project that provided comprehensive physical activity education. This is counter to usual care, which is more likely reflected in Group 2’s survey responses. |
| **Themes:**   1. Importance of flexibility and individualisation of programs 2. Preferences for optimal program design differ, but continuity and follow-up should be emphasised   **Quotes:**   - “No, I think it [education] should all be given in that one in the beginning, but then and also having some handouts… it sort of really need to know from the beginning… what you what you've got to do and who you have to see and what you know your exercise is and you know if you have problems who do you contact?” – Patient Interview #3 [Round 2] - “Maybe [commence education] at the time that it is probable. When the specialist, from the GP, determine that it [lung cancer] is probable.” – Patient Interview #2 [Round 2] | *Other: at any time prior to surgery (n=2) | Group 1 were surveyed regarding specific logistical elements of the prototype program after workshop one. These preferences were integrated into the first intervention prototype and further explored during the second round of workshops. The qualitative findings of round two demonstrated good convergence with the survey findings, likely due to this iterative mixed-method data collection process. Workshop round 2 further expanded upon these findings. When elucidating their survey responses, overall participants felt that education should commence as close to diagnosis as possible and provided some pragmatic suggestions for achieving this (e.g., utilising pre-admission clinics) during the workshops which further expanded upon the quantitative findings. |
| **Themes:**   1. Importance of flexibility and individualisation of programs 2. Preferences for optimal program design differ, but continuity and follow-up should be emphasised   **Quotes:**  “If it's useful [inpatient education] something that patients feel it would be and it almost serves as a reminder of all of those education topics that were listed, the 10 or 12 topics, we could cover those sorts of things… And then you can talk to your nurse, you can do this. Just as a reminder thing, I think that's a great idea.” – Professional, Workshop #2 [Round #2] |  | Again, there was a good degree of convergence between these findings. During the second round of workshops, participants agreed that post-operative education should commence before hospital discharge. Workshop round 2 further expanded on these findings, and participants proposed that education should also occur after hospital discharge. |
| **Themes:**   1. Importance of flexibility and individualisation of programs 2. Preferences for optimal program design differ, but continuity and follow-up should be emphasised   **Quotes:**   - “Can I just say that I think 2 weeks or 2-4 weeks is too long, but that might’ve just been because I was in high dependency for the 12 days and when I got out, it really knocked me about.” – Patient, Workshop #3 [Round 2] - “I think 2-4 weeks from a physio and a return to exercise is probably not a terrible amount of time. But then I agree with [patient] that 2 weeks of not hearing from someone and you being left to your own devices I think is too long.” – Professional, Workshop 3 [Round 2] |  | Most respondents felt that exercise should commence immediately on hospital discharge. This had a relatively good degree of convergence with our qualitative findings, however, when exploring this further, a few areas of expansion were noted:   - Patients should be provided education to commence a graded home-exercise program independently immediately after hospital discharge - An initial appointment focusing on progressing exercises/commencing more intense exercise may be more appropriate to occur 1-4 weeks after hospital discharge.   Qualitative findings also provided areas of complementarity, whereby participants provided suggestions such as screening for and incorporating patient needs and preferences regarding the timing of the first follow-up appointment and implementing a flexible approach to the first follow-up session. |

**Supplementary Table 9: Proposed intervention prototype according to the TIDieR Checklist [9]**

|  | **Brief Name** | Empowering recovery: A co-designed intervention to transform care for operable lung cancer | | | | |
| --- | --- | --- | --- | --- | --- | --- |
|  | **Why** | **Goals:**   - To improve access to evidence-based rehabilitation for patients undergoing surgery for lung cancer - To build patient and caregiver confidence, skills, self-efficacy and independence regarding rehabilitation and symptom management - To build patient health literacy to facilitate self-management - To facilitate improved continuity of care across the continuum of lung cancer care and reduce feelings of abandonment/isolation from the hospital system after hospital discharge - To meet patient and caregiver’s needs relating to education and knowledge/health empowerment, and setting of realistic expectations   **Rationale/Theory informing prototype design:**   - A multi-modal intervention co-designed with stakeholders, underpinned by behaviour change theory and evidence-based lung cancer exercise guidelines - To support environmental restructuring to facilitate enhanced access to support, monitoring and rehabilitation for patients with operable lung cancer - Underpinned by elements of training, education and enablement to support patient skill development to participate in exercise and self-management, self-efficacy and mental health | | | | |
|  | **What** | **Who Provided** | **How** | **Where** | **When and How Much** | **Tailoring** |
| **Pre-Operative** | **Screening**   - Comprehensive screening of: - Mobility - Exercise capacity - Respiratory status/symptoms - Other symptoms (e.g., fatigue, dyspnoea) - Current physical activity levels - Social supports - Functional independence - Smoking status - Mental health - Self-efficacy - Health and digital literacy - Utilising a screening template proforma incorporating the above elements via clinician-administered screening and patient-reported outcome measures | A central contact, aiming for continuity throughout the entire intervention (ideally physiotherapist or lung cancer nurse) with upskilling as needed in exercise prescription, education, and approaches to self-management | - One-on-one - Face-to-face* or remotely via telehealth (video and/or phone depending on digital and health literacy) | In hospital or remotely | - As close to diagnosis as possible - A once-off 60-minute initial appointment with the opportunity for an additional follow-up to ask questions if indicated - Patients may choose to access and refer to educational resources independently at any time | - An in-depth assessment where clinically indicated, occurring at another time before surgery either face-to-face or remotely pending patient availability and preference - Referral for additional multidisciplinary support (e.g., dietician, psychologist) external to the program where clinically indicated |
|  | **Education**   - Initial 1:1 education session on: - Diagnosis and prognosis - What to expect during hospital admission - Potential symptoms/side effects and management - Potential post-operative complications and management - Typical recovery journey and timeframes - Importance of physical activity and ‘fitness for surgery’ - Post-operative rehabilitation plan - Management of co-morbidities - Accounts of past patient experiences - Provision of physical handouts at a minimum, and if appropriate provide access to an online educational module (clinicians to determine digital literacy/appropriateness) reiterating and expanding on the above concepts, including written education and video-based content |  | - One-on-one - Face-to-face* or remotely - Reinforced via handouts and/or online module |  |  | - Opportunity for an additional 30-minute follow-up session for questions either face-to-face or remotely pending patient preference - Inclusion of caregivers during initial session - Additional caregiver education on how to empower, support and monitor loved ones’ recovery provided during the initial 60-minute session - Additional educational components covered during in-person education and reinforced via additional materials (pamphlets and online modules) were required e.g., neo/adjuvant treatment, smoking cessation, etc. - Mode of education delivery |
|  | **Exercise**   - Individualised aerobic and resistance training exercise prescription and goal setting based on patient preferences and physical capability - Provision of physical handouts containing: - Visual demonstrations of exercises - Written descriptions of exercises - Education on safety, intensity and self-monitoring (e.g., HR, BORG scale and RPE) - And if appropriate provide access to videos/online modules demonstrating prescribed exercises (clinicians to determine digital literacy/appropriateness) and reiterating education as above - Patient to participate in independent home exercise program as supported by the above resources |  | - One-on-one - First session prescribed face-to-face* or remotely - Prescribed exercises and technique reinforced via handouts and/or videos via online module | - First session to occur during the above screening/education session - Ongoing independent home-based exercise | Personalised, goal to reach evidence-based exercise guideline of 150-300 minutes of moderate-intensity exercise and >2 days of moderate-intensity resistance training per week completed until the day of hospital admission | - Personalised exercise prescription and dosage - Referral to formal, supervised program e.g., pulmonary rehabilitation based on patient preferences and needs - Ongoing, regular supervised exercise via Telehealth video or remote phone follow-up to monitor progress and motivation at an individualised interval pending patient needs and preferences - Provision of exercise diary and activity monitors based on patient preference to support motivation and adherence - Provision of exercise equipment (e.g., TheraBand or weights) if required |
| **Hospital Discharge** | **Screening**   - Comprehensive ‘check-in’ during inpatient stay focussed on: post-operative symptoms/impairments/activity restrictions, social supports, level of engagement with provided education resources, presence of ongoing knowledge gaps, exercise behaviours prior to admission (including barriers), self-efficacy, plans for exercise post-discharge, and preferences for ongoing follow-up. - Guided by a ‘checklist’ proforma incorporating the above elements, administered by clinician - Provision of a physical patient-version of ‘checklist’ completed asynchronously |  | - One-on-one - Synchronous face-to-face whilst in the acute hospital (before hospital discharge) with opportunity to complete ‘'homework’ tasks independently on the ‘checklist’ and reading of education resources | In hospital (at bedside or ward meeting room) | - - 24-48 hours before hospital discharge   - 30–45-minute session | - Opportunity for an additional 20-30-minute follow-up session for questions or additional screening before discharge |
|  | **Education**   - Reinforcement and redirection towards previously provided education and materials |  |  |  |  | - Individualisation of reinforced education based on existence of ongoing knowledge gaps |
|  | **Exercise**   - Reinforcement of previously provided education - Support to recommence safe activity - Prescription of upper-limb and thoracic spine mobility exercises (as per standard care) and education on graduated return to previous activity (e.g., walking) - Provision of physical handouts demonstrating prescribed exercises: - Visual demonstrations of exercises - Written descriptions of exercises - Education on safety, intensity and self-monitoring (e.g., HR, BORG scale and RPE) - And if appropriate provide access to videos/online modules demonstrating prescribed exercises (clinicians to determine digital literacy/appropriateness) and reiterating education as above |  |  |  |  | - Screen for preferences for mode of ongoing follow-up - Screen for preferences for timing of initial follow-up - Referral to formal, supervised program e.g., pulmonary rehabilitation based on patient preferences and needs |
| **Long-Term Recovery** | **Screening**   - Comprehensive screening/check-in guided by the same ‘checklist’ as above - Personalised collaborative goal setting utilising motivational interviewing techniques |  | - One-on-one - Face-to-face* or remotely | In patients’ home environment or remotely | - 2-4 weeks after hospital discharge - A once-off 60-minute initial appointment - Patients may choose to access and refer to educational resources independently at any time | - Session timing and location/delivery mode - Referral for additional multidisciplinary support (e.g., dietician, psychologist) external to the program where clinically indicated |
|  | **Education**   - Reinforcement and redirection towards previously provided education and materials |  | - One-on-one - Face-to-face* or remotely - Reinforced via handouts and/or online module |  |  | - Individualisation of reinforced education based on the existence of ongoing knowledge gaps |
|  | **Exercise**   - Individualised aerobic and resistance training exercise prescription and goal setting based on patient preferences and physical capability - Provision of physical handouts demonstrating prescribed exercises: - Visual demonstrations of exercises - Written descriptions of exercises - Education on safety, intensity and self-monitoring (e.g., HR, BORG scale and RPE) - And if appropriate provide access to videos/online modules demonstrating prescribed exercises (clinicians to determine digital literacy/appropriateness) and reiterating education as above - Patient to participate in independent home exercise program as supported by the above resources |  | - One-on-one - First session prescribed face-to-face* or remotely - Prescribed exercises and technique reinforced via handouts and/or videos via online module | - First session to occur during the above screening/education session - Ongoing independent home-based exercise | - Initial prescription session during above 60-minute appointment - Remote follow-up (via video or phone Telehealth) ^A^ at an interval set in collaboration with patient if required - Length of follow-up determined by patient progress and goals   Personalised, goal to reach evidence-based exercise guideline of 150-300 minutes of moderate-intensity exercise and >2 days of resistance training per week | - Mode, length and frequency of ongoing follow-up (with appreciation that some patients may only require education and initial exercise prescription i.e., no ongoing follow-up) - Incorporation of manual therapy for pain management - Provision of exercise diary and activity monitors based on patient preference to support motivation and adherence - Provision of exercise equipment (e.g., TheraBand or weights) if required - Referral to formal, supervised program e.g., pulmonary rehabilitation based on patient preferences and needs |
| **Across All Timepoints** | **Mental Health Support**   - Incorporated within all screenings using quick validated measures such as PHQ-9 or HADs to screen for depression and anxiety - Education on local services (e.g. mental health programs and/or accessing support through general practitioner) and self-management (including meditation, mindfulness resources) with a focus on cognitive behavioural approaches which could be delivered by clinicians upskilled in this area (e.g. physiotherapist, clinical nurse) due to accessibility gaps in psychology incorporated within education and education handouts/modules |  | - One-on-one - Face-to-face* or remotely - Education on techniques and available supports reinforced via handouts and/or online module | As above | From diagnosis, available alongside program | - Referral to external support where specific psychological support is required (e.g., via hospital psychological service or General Practitioner) - Referral or linkage with external peer-support programs depending on individual preference |
|  | **Hotline Phone Number**   - Access to intervention provider via phone for questions, symptoms and support |  | Remote support | Via phone | - Available from program commencement until discharge, available alongside program - Patient-initiated contact |  |
| *Preference for face-to-face where feasible  ^A^ Based on patient preference where feasible, aim for video if appropriate (i.e., if adequate digital access and literacy)  **Abbreviations:** HR = heart rate; RPE = Rate of perceived exertion, PHQ = Patient Health Questionnaire, HADS = Hospital Anxiety and Depression Scale | | | | | | |

**References**

1. The Point of Care Foundation. *EBCD: Experience-based co-design toolkit*. 2013; Available from: <https://www.pointofcarefoundation.org.uk/resource/experience-based-co-design-ebcd-toolkit/>.

2. da Silva, A.A., et al., *'How Do I Test the Waters? How Do I Go Forward?': Co-designing a Supportive Pathway after Critical Illness.* Annals of the American Thoracic Society, 2024(2325-6621 (Electronic)).

3. Tang, C.Y., et al., *Adopting a collaborative approach in developing a prehabilitation program for patients with prostate cancer utilising experience-based co-design methodology.* Supportive Care in Cancer, 2020. **28**(11): p. 5195-5202.

4. Michie, S., M.M. van Stralen, and R. West, *The behaviour change wheel: a new method for characterising and designing behaviour change interventions.* Implementation science : IS, 2011. **6**: p. 42-42.

5. Cane, J., D. O'Connor, and S. Michie, *Validation of the theoretical domains framework for use in behaviour change and implementation research.* Implementation science : IS, 2012. **7**: p. 37-37.

6. Australian Government Department of Health and Aged Care. *Health Workforce Locator*. 2024; Available from: <https://www.health.gov.au/resources/apps-and-tools/health-workforce-locator>.

7. Lumivero, *NVivo (Version 14)*. 2023: <www.lumivero.com>.

8. Michie, S., L. Atkins, and R. West, *The Behaviour Change Wheel: A Guide to Designing Interventions*. Vol. 1. 2014, Great Britain: Silverback Publishing.

9. Hoffmann, T.C., et al., *Better reporting of interventions: template for intervention description and replication (TIDieR) checklist and guide.* BMJ, 2014. **348**.

10. Skamagki, G., et al., *The concept of integration in mixed methods research: a step-by-step guide using an example study in physiotherapy.* Physiotherapy Theory and Practice, 2024. **40**(2): p. 197-204.

11. Tong, A., P. Sainsbury, and J. Craig, *Consolidated criteria for reporting qualitative research (COREQ): a 32-item checklist for interviews and focus groups.* International Journal for Quality in Health Care, 2007. **19**(6): p. 349-357.

12. Staniszewska, S., et al., *GRIPP2 reporting checklists: tools to improve reporting of patient and public involvement in research.* BMJ, 2017. **358**: p. j3453.

13. Duncan, E., et al., *Guidance for reporting intervention development studies in health research (GUIDED): an evidence-based consensus study.* BMJ Open, 2020. **10**(4): p. e033516.
